# Effects of Distal Mutations on the Structure, Dynamics and Catalysis of Human Monoacylglycerol Lipase

**DOI:** 10.1038/s41598-017-19135-7

**Published:** 2018-01-29

**Authors:** Sergiy Tyukhtenko, Girija Rajarshi, Ioannis Karageorgos, Nikolai Zvonok, Elyssia S. Gallagher, Hongwei Huang, Kiran Vemuri, Jeffrey W. Hudgens, Xiaoyu Ma, Mahmoud L. Nasr, Spiro Pavlopoulos, Alexandros Makriyannis

**Affiliations:** 1 Center for Drug Discovery and Departments of Pharmaceutical Sciences and Chemistry and Chemical Biology, Northeastern University, Boston, Massachusetts, 02115-5000 USA; 2 BioProcess Measurements Group, Biomolecular Measurement Division, National Institute of Standards & Technology, Rockville, MD 20850 USA; 3 Institute for Bioscience and Biotechnology Research, 9600 Gudelsky Drive, Rockville, MD 20850 USA; 4 Department of Chemistry and Biochemistry, Baylor University, Waco, TX 76798 USA; 5 Schrödinger, 222 Third Street Suite 2230, Cambridge, MA 02142 USA; 6 Department of Biological Chemistry and Molecular Pharmacology, Harvard Medical School, Boston, Massachusetts, 02115 USA; 7 Present Address: Dassault Systèmes, 9 Industrial Road, Milford, MA 01757 USA

## Abstract

An understanding of how conformational dynamics modulates function and catalysis of human monoacylglycerol lipase (hMGL), an important pharmaceutical target, can facilitate the development of novel ligands with potential therapeutic value. Here, we report the discovery and characterization of an allosteric, regulatory hMGL site comprised of residues Trp-289 and Leu-232 that reside over 18 Å away from the catalytic triad. These residues were identified as critical mediators of long-range communication and as important contributors to the integrity of the hMGL structure. Nonconservative replacements of Trp-289 or Leu-232 triggered concerted motions of structurally distinct regions with a significant conformational shift toward inactive states and dramatic loss in catalytic efficiency of the enzyme. Using a multimethod approach, we show that the dynamically relevant Trp-289 and Leu-232 residues serve as communication hubs within an allosteric protein network that controls signal propagation to the active site, and thus, regulates active-inactive interconversion of hMGL. Our findings provide new insights into the mechanism of allosteric regulation of lipase activity, in general, and may provide alternative drug design possibilities.

## Introduction

Inhibition of brain endocannabinoid hydrolase hMGL enhances endocannabinoid levels, indirectly activates cannabinoid receptors, and modulates cannabinergic signaling. Understanding the molecular basis of monoacylglycerol lipase (MGL) function is critical for the development of novel, selective inhibitors of potential therapeutic value. MGLs play an important role in lipid catabolism by catalyzing specifically the hydrolysis of monoacylglycerol into free fatty acid and glycerol. MGL serves specialized metabolic functions in different cells and tissues^1^. In the central nervous system this enzyme hydrolyses 2-arachidonoylglycerol (2-AG), an agonist of cannabinoid receptor type 1 (CBR1)^2,3^, the structure of which was recently determined^4^. The endocannabinoid system is involved in the regulation of various physiological and pathological processes related to the nervous system^5^. MGL is recognized as the major regulator of 2-AG levels in the brain and of CBR1-dependent cellular and behavioral responses^6–10^. The therapeutic potential of MGL inhibitors for the treatment of pain and inflammation, depression, nausea, neurodegeneration, precipitated opioid or cannabis withdrawal responses, and cancer pathogenicity, due to MGL blockade, has been investigated^11–19^.

MGLs belong to a superfamily of α/β hydrolase enzymes that utilize a nucleophile-histidine-aspartate catalytic triad in cooperation with an oxyanion hole for catalysis. For the lipases and esterases the nucleophile is a serine residue. The 3D hMGL crystal structures of apo hMGL in the open forms (PDB:3HJU and 3JW8) are reported^20,21^. The crystal structure of hMGL, complexed with the irreversible inhibitor SAR-629^21^, revealed holo hMGL in an open conformation, while the crystal structure of hMGL complexed with the reversible inhibitor Compound-1 (PDB:3PE6) exhibited holo hMGL in a closed conformation^22^. Collectively, these structures revealed the existence of a functionally important subdomain referred to as the “lid” (residues 151–225) that controls access to the active site of the enzyme.

The crystal structures of apo and holo bacterial MGL (bMGL) from *Bacillus* sp^23^. as well as an apo mutant (D196N) and this mutant in complex with several substrate analogs^24^ showed conservation of the overall lid architecture between hMGL and bMGL. Interestingly, these structures capture different conformations of the lid region. Specifically, the structure of the apo bMGL mutant (D196N), which contains six different chains in the asymmetric unit, revealed sampling of “super-opened” and partially restricted conformations. Comprehensive analyses of all bMGL structures revealed a high degree of conformational plasticity of the lid domain, suggesting the existence of a stochastic equilibrium between open and restricted lid conformations. In contrast, a recently published structure of authentic bMGL isolated from *Bacillus* sp. H257 appears to be in a closed conformation^25^.

We have demonstrated that hMGL exists in solution in a dynamic equilibrium between its open and closed forms, which is slow on the NMR time scale. Thus, hMGL is a rare example of a conformationally flexible lipase that can be quantified^26^. We found a direct link between these conformational changes and their impact on hMGL catalytic activity. Understanding the molecular basis of MGL function is critical for the development of novel, highly selective inhibitors. Previously, we have characterized recombinant hMGL and its interactions with inhibitors by various biochemical and biophysical methods^27–30^.

A recent trend towards improving compound selectivity in drug discovery is the strategy of targeting allosteric sites^31^ rather than the active site directly. Targeting allosteric sites offers the opportunity to improve targeting for the chosen protein. Allosteric sites can be more accessible than buried active sites, can function in concert with direct active site regulators, do not interfere with endogenous regulators, and can serve as activable modules^31^. However, no such allosteric sites and pairwise interactions responsible for propagation of conformational changes from distal sites to the hMGL active site are currently known.

Our aim is to identify intramolecular communication between residue pairs and to characterize allosteric coupling between distal sites that are related to hMGL function. Although a number of hMGL X-ray structures are available, two major complications in the identification of potential allosteric sites and pairwise interactions exist. First, only a small subset of protein residues are believed to be involved in the formation of physically coupled interactions that link remote functional sites in the 3D structure. Second, detailed examinations of 3D structures do not straightforwardly reveal the diverse allosteric interactions among distal and active sites^32^. Studies of a serine protease found that the distal Trp-23 residue is important for catalysis, as well as substrate binding^33,34^. It is also well known that tryptophan residues with their manifold properties often play an important role in lipase architecture and function^35,36^. We therefore focused our attention on tryptophan residues.

For the present study, we used a mutational structure perturbation approach to reveal long-range communication and the presence of conformational switches in the interior of the hMGL enzyme. Structural, dynamic, and functional responses to conservative and nonconservative mutations were observed and characterized. Specifically, we have found that structural excitations at Trp-289 and Leu-232 lead to conformational changes and concerted motions of structurally distinct regions. Experimental insight into the mechanism of inter-residue interactions within a communication network was achieved by combination of site-directed mutagenesis, kinetics, nuclear magnetic resonance (NMR) and circular dichroism (CD) spectroscopy, hydrogen deuterium exchange mass spectrometry (HDX-MS) and molecular dynamic (MD) simulations. Using this multimethod approach, we demonstrate that the effects of a single point mutation at a specific distal site of hMGL are transduced >18 Å to the active site and to other remote regions, suggesting allosteric regulation of hMGL function.

## Results

### Selection of Distal Residues for Substitution

**Figure 1:**
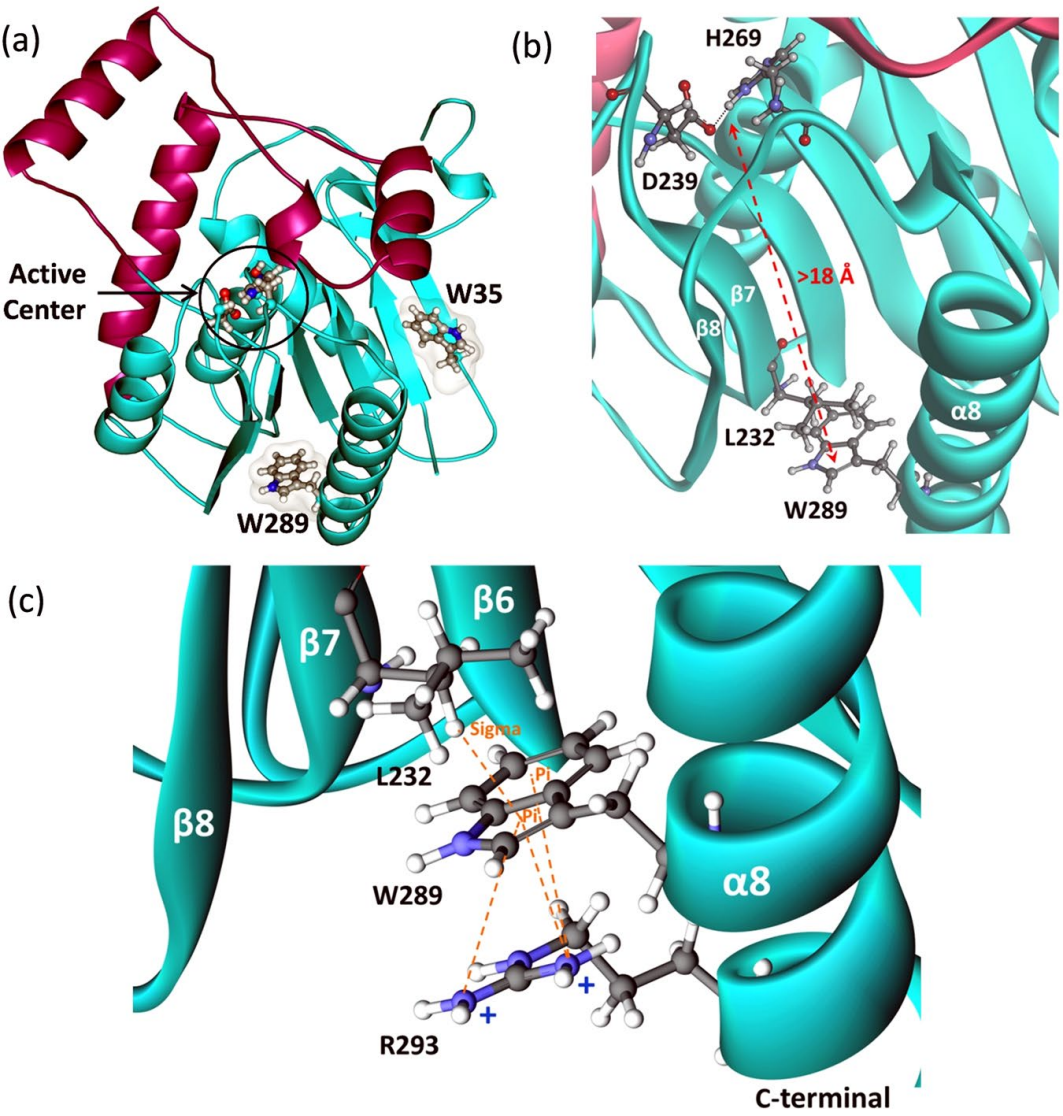
(**a**) The overall hMGL 3D structure (PDB ID:3HJU) showing the location of two conserved Trp-35 and Trp-289 residues relative to the active site of the enzyme. The lid domain is highlighted in magenta. (**b**) Fragment of this structure zoomed in to provide a view of the distal residues Trp-289 and Leu-232 that reside more than 18 Å away from the catalytic triad residues. (**c**) Close-up view of the identified remote site. Interactions between side-chains of Trp-289, Leu-232 and Arg-293 are highlighted.

hMGL contains only two tryptophan residues, which reside at positions 35 and 289 (Fig. 1a). While the MGL sequence displays no extensive homology to other mammalian proteins, both tryptophan residues are conserved in distantly related bacterial esterases and haloperoxidases^37^. Tryptophan side chains may play an important structural role in stabilizing the interactions of the β sheet with the helices on one side, similar to that observed for other lipases^35,36^. Trp-35 is located in the N-terminal domain in the exposed beta sheet (Fig. 1a), whereas Trp-289 is located in the C-terminal of helix 8, which is greater than 18 Å away from the catalytic center (Fig. 1b). The bulky hydrophobic indole ring of Trp-289 is positioned exactly between strand β7 and helix α8. Since the catalytic Asp-239 is anchored on the loop after β7 and the catalytic His-269 is anchored on the loop between β8 and α8, we hypothesized that structural perturbations at Trp-289 may change the relative distances among the catalytic triad residues (Fig. 1b). Single substitutions at both these positions were generated: Trp-35 was replaced with Ala and Trp-289 with Ala/Leu/Phe. Trp-289 demonstrates extensive involvement in the cation-π interactions with Arg-293 from α8 and C-H∙∙∙π interaction with Leu-232 from β7 (Fig. 1c). To examine the importance of hydrophobic, aromatic and electrostatic interactions among Trp-289 and the side chains of Arg-293 and Leu-232, we mutated each of these residues individually to Ala and Gly, respectively, thereby eliminating these interactions. Replacement of Leu-232 with a small Gly residue was chosen to minimize the strength of hydrophobic interactions that may integrate β7 with α8 secondary structural elements in the tertiary structure of hMGL (Fig. 1).

### Expression, Purification and Molecular Characterization

All the mutants used in this study were constructed based on a previously described soluble hMGL (sol-hMGL) variant that has two leucines substituted for two serines (L169S/L176S). These substitutions confer sufficient aqueous solubility to achieve desired concentrations for NMR experiments^26^. Additional single-point mutations were introduced in the sol-hMGL construct. The HDX-MS studies were performed with the wild type hMGL and a wild type based W289L mutation. To assess the effects of point substitutions on enzymatic structure and function, we first evaluated the expression and purification of hMGL mutants. All the hMGL mutants were expressed and purified as previously reported^26^. Expression of the W289A mutant was very low, as evidenced by the SDS-PAGE data, suggesting that this mutation has a severe effect on protein expression. All other constructs expressed with yields similar to that of sol-hMGL. SDS-PAGE and size-exclusion chromatography (data not shown) were employed to confirm the purity and oligomeric state of the protein preparations (Supplementary Fig. S1). Sol-hMGL and the mutants were of high purity and eluted primarily as a monomer.

### Impact of Distal Amino Acid Substitutions on hMGL Catalysis and Identification of Essential Residues

To gain insight into the catalytic chemistry and function of hMGL, we sought to identify essential residues that are remote to the catalytic site of the enzyme. These hypomutable regions with putative essential residues were localized on the bases of a literature search^33,34^. Nonconservative single point substitutions revealed that the W35A mutation has a minimal effect on the enzymatic activity, whereas the W289L mutation demonstrated a dramatic 10^5^-fold loss in the catalytic efficiency (Table 1 and Supplementary Fig. S2). However, the conservative substitution of the same Trp-289 with an aromatic residue Phe had almost no effect on hMGL hydrolytic performance. Although, the enzymatic affinity *(K*_m_) showed a slight decrease to (46 ± 7) µM, its catalytic efficiency was virtually intact. This suggests that specific, through planar stacking interactions of aromatic residues, such as Trp and Phe, at this site are likely involved in regulation of hMGL activity.

**Table 1:** Steady state kinetics parameters of sol-hMGL and the mutants based on the hydrolysis of endogenous substrate 2-AG.

Protein	*K*_m_ (µM)	*k*_cat_ (sec^−1^)	Catalytic Efficiency	Fold Decrease
Sol-hMGL	22 ± 4	(5.7 ± 0.2) × 10^5^	2.5 × 10^4^	
W35A	43 ± 4	(2.5 ± 0.7) × 10^5^	6 × 10^3^	4
W289L	22 ± 2	1.06	4.7 × 10^–2^	5.2 × 10^5^
W289F	46 ± 7	(1.2 ± 0.01) × 10^6^	2.6 × 10^4^	0.9
R293A	57 ± 7	(5.8 ± 0.2) × 10^5^	9.9 × 10^3^	2.5
L232G	16 ± 3	17 ± 0.9	1.04	2.4 × 10^4^

Each measured value is listed with the corresponding 1σ uncertainty.

Of the two tryptophans, only Trp-289 was shown to play a key role in enzymatic function. Only nonconservative substitution at position 289 indicated an essential role in the regulation of hMGL function, opening new possibilities for further exploration in the region near Trp-289. Therefore, we shifted our focus onto residue substitutions that explore interactions with the Trp-289 side chain. The R293A and L232G mutants allowed us to examine these pairwise interactions. Remarkably, the consequences of Arg-293 replacement on 2-AG hydrolysis were negligible. The enzymatic affinity was found to be somewhat higher than that found for sol-hMGL (*K*_m_ = (57 ± 7) µM), while the turnover number (*k*_cat_) was unchanged (Table 1). In contrast, the kinetic behavior of the L232G mutant was severely compromised similar to that observed in the W289L mutant. The enzymatic affinity of the L232G mutant for 2-AG was similar to that of sol-hMGL (*K*_m_ = (16 ± 3) µM), however, the catalytic efficiency dropped by four orders of magnitude (2.4 × 10^4^) (Table 1). In summary, examination of the kinetic data for distal mutations revealed an essential role of Trp-289 and Leu-232 residues in hMGL catalysis.

### Downfield ^1^H NMR Spectral Pattern of hMGL as a Probe for Conformational Analysis

**Figure 2:**
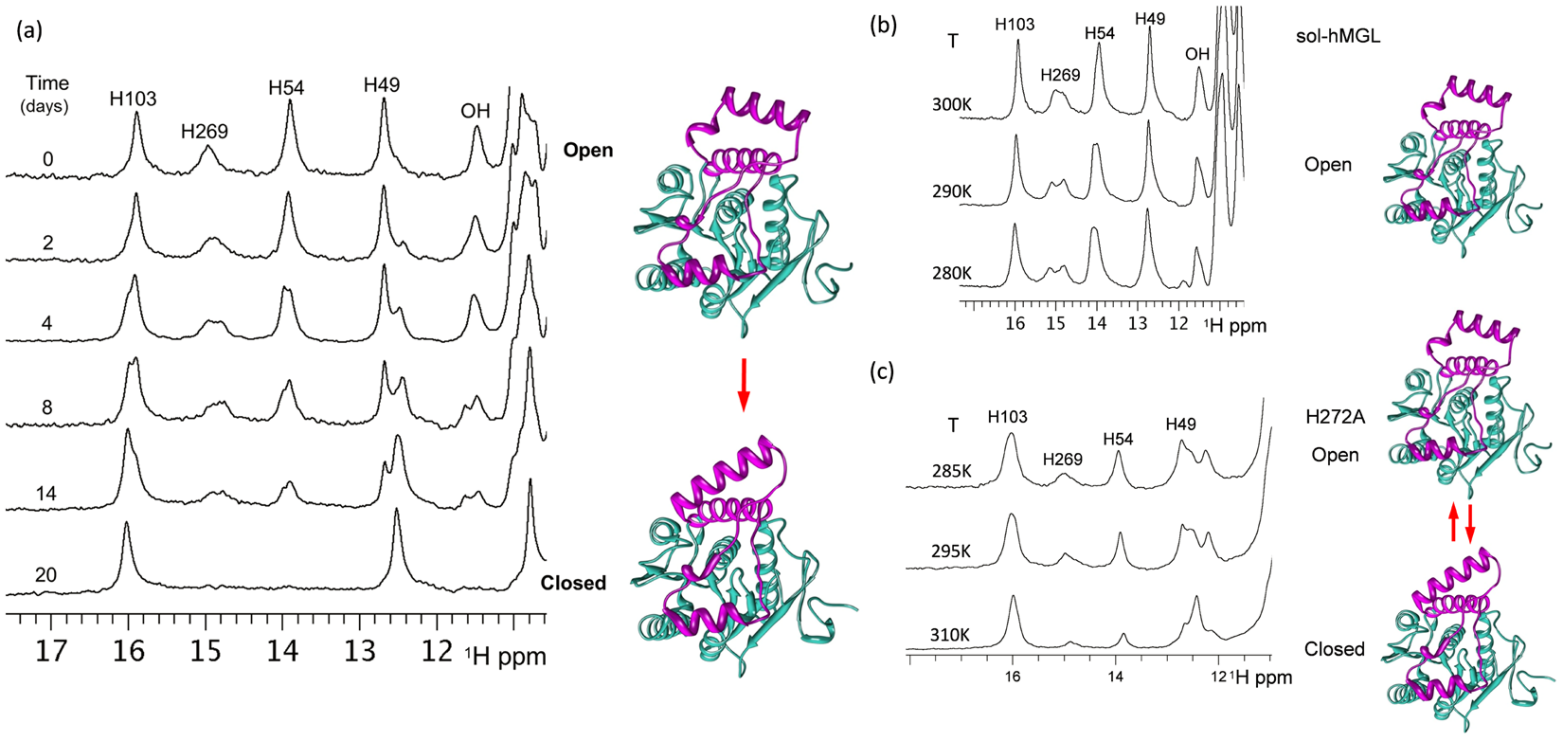
(**a**) Real-time NMR spectra demonstrating spontaneous, sequential interconversions among different lid domain conformations in the transition pathway between two extreme conformations: open and closed (T = 310 K, pH 7.4). Variable temperature NMR experiments demonstrating (**b**) temperature effect on the rate of exchange between open conformations of sol-hMGL and (**c**) effect of temperature on the distribution of open-closed conformers for the H272A mutant (pH 7.4).

In earlier work we demonstrated that the downfield region of 1D ^1^H NMR spectra (11–16 ppm) of hMGL in open form displays five distinct and well separated peaks, corresponding to the side chains involved in intramolecular hydrogen bonding networks^26^. Importantly, these peaks are highly sensitive to the local and global (open/closed) conformational interconversions of hMGL. The resonances (Fig. 2a) at 15.9, 14.9, 13.9 and 12.8 ppm have been assigned to the His-103, His-269 (catalytic histidine), His-54 and His-49, respectively^26^. The resonance at 11.5 ppm belongs to an unassigned OH group. These peaks represent the downfield ^1^H NMR spectral pattern of the active (open) hMGL form. Our wild type and sol-hMGL protein preparation protocol typically yields enzyme in the open (active) conformation, which is stable for several weeks at room temperature. In the absence of detergents the open conformer slowly undergoes a spontaneous transition to the closed (inactive) state, suggesting that in water the closed conformational state of hMGL is preferred. The spectral pattern of the closed form differs significantly, with the absence of His-54 and His-269 resonances from their initial positions in the spectra of the open form. The absence of the His-54 resonance at 13.9 ppm in the closed conformation is due to it shifting well outside of the downfield region as a result of breaking the hydrogen bond between His-54 and Asp-197. His-54–Asp197 hydrogen bonding integrates the hMGL core with the lid domain, and this interaction is likely one of the crucial factors controlling the lid domain mobility. We have demonstrated that dynamic breaking and forming of this bond are directly associated with the open-to-closed and closed-to-open interconversions (26). In concert with the His-54 resonance, the catalytic triad His-269 resonance can also disappear and reappear, reflecting reversible conformational changes of the catalytic triad and synchronized movements of the lid domain. In contrast to the dynamic bonding of His-54–Asp197, the His-269–Asp-239 hydrogen bonding is intact in both open and closed conformations. In the closed conformation the His-269 resonance exhibits a significant upfield shift and broadening. As a result, the closed conformation of hMGL is characterized by a reduced number of resonances in the downfield part of NMR spectra. This allows the simultaneous NMR detection of both conformers in solutions as well as monitoring of time-dependent lid domain-closing events. These distinct NMR patterns enable unambiguous recognition of individual conformers in mixtures (Fig. 2) and quantification of the conformational equilibrium affected by the physical environment, mutations, and ligand binding. Figure 2a demonstrates the detection of spontaneous transitions from open to closed states in aqueous solvent (in the absence of an interface) on the bases of downfield NMR resonances. In fact, Fig. 2a presents a series of real-time NMR spectra, measured over 20 days, evidencing directly the relatively slow conformational changes due to lid domain movements. Such lid domain movements can affect catalytic turnover. Time course (Fig. 2a) along with temperature dependent NMR experiments (Fig. 2b) reveal broadening and splitting of individual peaks along the open-to-closed conformational transition pathway of hMGL. Detailed analyses of these spectra suggest the presence of multiple, discrete, open conformations that sequentially interconvert into the discrete closed conformations. These interconversions are slow to intermediate on the NMR time scale, which allows direct detection of intermediate conformations in the path. Thus, the ensemble of open dynamical conformations undergoes a population shift, redistributing toward those states favored by the conditions and affecting hMGL function.

Substitution of H272A in hMGL represents an example of a reversible interconversion shifting the delicate equilibrium towards closed conformations at T = 310 K (Fig. 2c). In turn, temperature variation also affects the population distribution. The NMR spectra clearly show a gradual increase in the peak intensities of His-54 and His-269, indicating an increase in the population of open forms with decreasing temperature. The 1D ^1^H NMR results provide the experimental evidence for a high degree of conformational heterogeneity of hMGL in solutions and strongly suggest that this heterogeneity is due to the conformational plasticity of the enzyme’s lid domain. Overall, 1D ^1^H NMR downfield resonances of hMGL can serve as a unique tool to monitor conformational changes induced by mutations and the physical environment.

### Evaluation of the Effect of a Distant Single Point Mutation on the Stability of the sol-hMGL Fold

To ensure that the W289L, W289F and L232G mutations do not cause a significant degree of unfolding of the hMGL enzyme, CD and NMR spectroscopy experiments were conducted.

**Figure 3:**
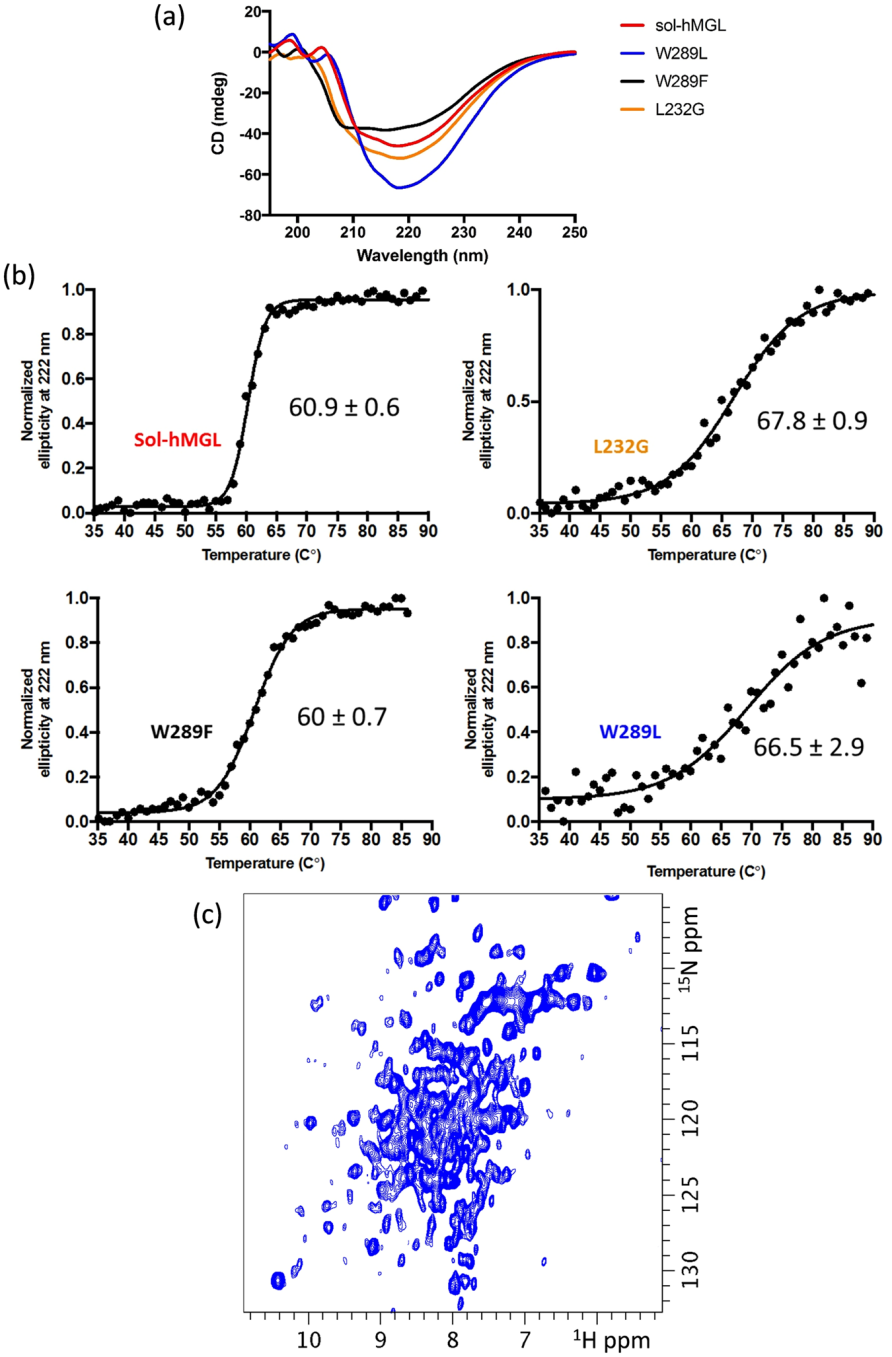
(**a**) Comparison of the far-UV CD spectra of sol-hMGL and mutant enzymes. The protein concentrations were ~10 µM. (**b**) Thermal denaturation curves of sol-hMGL and mutants. Enzymes were subjected to a temperature gradient in 20 mM sodium phosphate, 100 mM NaCl, 1 mM DTT buffer, pH 7.4, and unfolding was followed by monitoring the CD signal at 222 nm. Data points are shown in black circles and the sigmoidal fits used to determine Tm. (**c**) Folding of W289L mutant assessed by two-dimensional ^1^H-^15^N HSQC NMR spectroscopy. Spectrum was acquired for uniformly ^15^N isotope-labeled sample at 310 K, pH 7.4, C = 150 µM.

CD experiments in the far-UV region (190–260 nm) were performed to compare the secondary structures and stability of sol-hMGL and the mutants (Fig. 3a). The CD spectrum of sol-hMGL is characteristic of a structure containing β-sheet and α-helices, with two minima at 209 and 222 nm. The CD spectra clearly show that all mutants have distinct secondary structures and that there is no sign of a significant amount of random coil structure.

A difference also was noticed in the behavior of the mutants to thermal denaturation. Thermal unfolding curves were obtained by monitoring the change in the negative CD signal at 222 nm as a function of temperature (Fig. 3b). Sol-hMGL demonstrated a melting temperature of 60 °C with a high level of cooperativity. The mutants L232G and W289L had a modest increase in melting temperature, whereas the mutant W289F had a melting temperature similar to sol-hMGL. The slopes of the melting curves of the mutants were not as steep as observed for sol-hMGL, indicating a loss of cooperative unfolding. Overall, CD spectroscopy data unambiguously show that W289L and L232G mutations do not cause loss of hMGL secondary structure. Control CD spectra of mutants in the presence of 8 M urea demonstrated the expected loss of secondary structure (Supplementary Fig. S3).

The similarity of secondary and tertiary structure of mutants and sol-hMGL was also assessed by 1D ^1^H NMR spectroscopy. As can be seen in Fig S4, similar to the sol-hMGL in the closed state, W289L and L232G spectra demonstrate the appearance of several peaks in the downfield region (17.5 to 9.7 ppm) as well as in the very upfield region (0.7 to −0.85 ppm). The high similarity in peak dispersion suggests that these mutants are still folded into structures resembling sol-hMGL. Tertiary fold stability of W289L and L232G can be evaluated using 2D ^1^H-^15^N heteronuclear single quantum correlation (HSQC) spectra. These spectra were recorded at different temperatures to assess the degree of an amide resonances dispersion and line broadening. The cross peaks of W289L at 310 K (Fig. 3c) are well dispersed, indicating the presence of a well-defined secondary structure. However, it can be seen that these peaks are severely broadened and poorly resolved. In fact we observed severe peak shape heterogeneity characteristic of the intermediate to slow chemical exchange rates between different conformations. The similar peak shape heterogeneity is also present in the ^1^H-^15^N HSQC spectra of L232G (data not shown). This type of spectra is indicative of a molten globule state, where secondary structural elements are presented shown by wide dispersion of an amide peaks, but tertiary structure is compromised and unstable. Thus, CD and NMR results indicate that no change occurs to the secondary fold due to nonconservative substitutions at positions 232 and 289. However, a lack of fixed tertiary interactions results in a conformational shift toward the ensemble of fluctuating structures.

### Effect of Distal Mutations on NMR Spectra and on Conformational Equilibria

**Figure 4:**
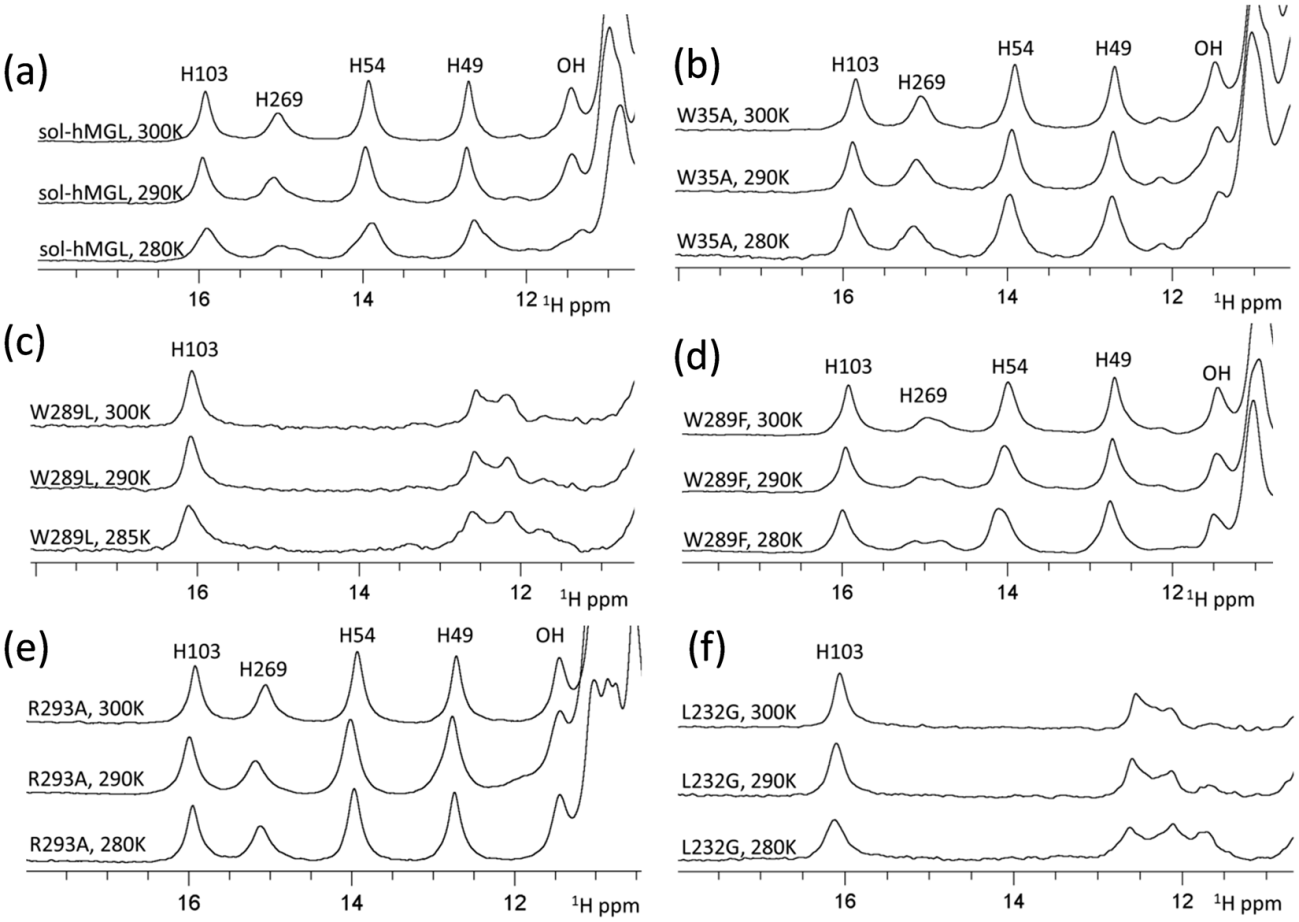
(**a**) The downfield ^1^H NMR spectra of sol-hMGL with temperature dependence behavior. The effect of single-point mutations and temperature for (**b**) W35A; (**c**) W289L; (**d**) W289F; (**e**) R293A and (**f**) L232G (pH 7.4).

Nonconservative substitution of Trp-35 did not substantially affect the catalytic efficiency of the enzyme. The downfield region of the ^1^H NMR spectrum of the W35A mutant (Fig. 4b) contains five distinct resonances, which are identical in chemical shifts and intensities to the pattern of the open conformer. The similarity (Fig. 4a,b) is evidence that the Trp-35 mutation prevents conformational transition to the closed forms. Furthermore, at all temperatures the number, position, and linewidths of peaks are almost identical to those of sol-hMGL, suggesting that open conformers are stable within the temperatures in the range, *T* = 285 K to 310 K. Thus, hMGL demonstrates a high tolerance to mutation at position 35, highlighting that it is not essential to the network of interactions modulating the enzyme’s function.

In contrast, the nonconservative substitution of Trp-289 correlates with a significant loss of catalytic efficiency, suggesting that it is an essential residue for long-range communication in hMGL. The downfield ^1^H NMR spectral pattern of the W289L mutant consists of two groups of signals (Fig. 4c). One group resides at 16 ppm (His-103 peak) and another group resides around ≈12 ppm (His-49 peak and others). Notably, the absences of His-269 and His-54 resonances underline the similarity of this pattern with that of the closed conformer. However, disappearance of these peaks is likely due to the conformational heterogeneity clearly observed in an amide region of 2D ^1^H-^15^N HSQC spectra. The greatly reduced catalytic efficiency in combination with increased conformational heterogeneity induced by W289L substitution strongly suggests an equilibrium shift towards the ensemble of very dynamic conformers that lack fixed tertiary interactions. The absence of temperature-dependent changes in NMR spectra (Fig. 4c) supports the irreversibility of observed shift and excludes the explanation of this transition based on open-closed equilibria. For reference, the H272S mutation shifts the equilibrium toward the closed conformations at physiological temperatures^26^; however, decreasing the temperature increases the intensity of His-269 and His-54 peaks, providing evidence for the reversibility of the equilibrium in this case. Thus, nonconservative substitution of Trp-289 induces an irreversible population shift of the conformational states toward species with native-like secondary structure that lack the native tertiary side-chain packing interactions.

In the case of a conservative substitution of Trp-289 with Phe, the downfield NMR spectral pattern (Fig. 4d) is similar to sol-hMGL, indicating that this mutation does not lead to a shift in population toward closed conformations. The similarity of spectra observed between *T* = 280 K and 300 K indicates the stability of the open forms. Congruently, the catalytic efficiency of the enzyme is not affected. Thus, hMGL tolerates a conservative substitution at position 289.

The hMGL crystal structure implies intensive inter-residue interactions between Trp-289 and Arg-293 side chains (Fig. 1c), potentially implicating this pairwise interaction with enzyme function. Discordant with this expectation, the enzyme tolerates a nonconservative substitution of Arg-293 with Ala, and the R293A mutant remains in open form between *T* = 280 K and 310 K (Fig. 4e). Congruently, the catalytic efficiency of this mutant is not compromised (Table 1), demonstrating that Arg-293 is not an essential residue for hMGL activity.

In contrast, the nonconservative L232G mutation results in nearly total loss of catalytic efficiency, which points out the essential role of pairwise interactions between Trp-289 and Leu-232 residues in hMGL. Remarkably, the downfield regions of NMR spectra for L232G (Fig. 4f) and W289L (Fig. 4c) are almost identical. Furthermore, spectra of both mutants exhibit no evidence for reversible temperature-dependent conformational changes similar to sol-hMGL. These observations are consistent with an irreversible shift of the conformational equilibrium toward an ensemble of very dynamic fluctuating structures. Thus, disruption of the interactions between Trp-289 and Leu-232 leads to a significant decrease of hMGL catalytic efficiency, presumably, through a conformational shift. The results underline the essential roles of two remote residues in regulation of enzyme function.

### Global Structural Changes of hMGL Due to Distal Local Perturbations

**Figure 5:**
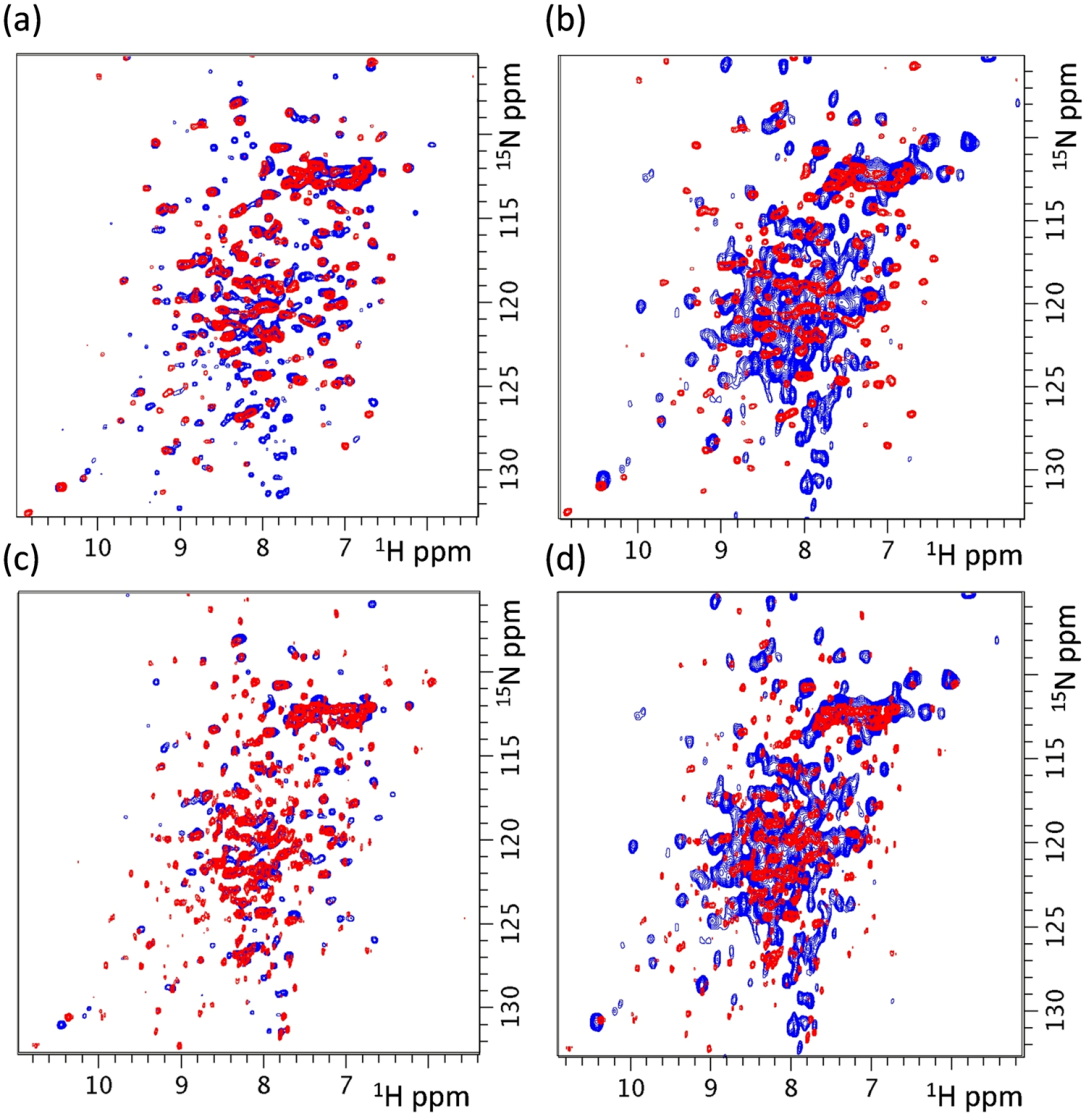
Superposition of two-dimensional ^1^H-^15^N HSQC spectra of ^15^N-labeled proteins (**a**) open sol-hMGL (red) and W289F (blue), (**b**) open sol-hMGL(red) and W289L (blue), (**c**) closed sol-hMGL (red) and W289F (blue) and (**d**) closed sol-hMGL (red) and W289L (blue) at 300 K, pH 7.4.

Superposition of 2D ^1^H-^15^N HSQC spectra of the open sol-hMGL form and active W289F mutant (Fig. 5a) clearly demonstrates that global conformational changes occur upon conservative substitution at position 289. It is noteworthy that the observed structural changes do not shift the conformational equilibrium toward a closed form, as evidenced by kinetic and downfield ^1^H NMR data. The similarity in the catalytic efficiencies of these constructs provides evidence that hMGL successfully tolerates this perturbation (Table 1).

Figure 5b displays the 2D ^1^H-^15^N HSQC spectrum of open sol-hMGL superimposed with the spectrum of the inactive W289L mutant. This superposition clearly shows the occurrence of global conformational changes induced by local perturbations at position 289. In addition, unlike the conservative substitution, the W289L mutation triggers a conformational shift toward significantly disordered conformers, as is evidenced by severe line shape heterogeneity in an amide region and the kinetic data for the W289L mutant. Superimposed 2D NMR spectra of closed sol-hMGL with active W289F and closed sol-hMGL with inactive W289L are presented in Fig. 5c,d to demonstrate the structural differences between mutants and closed sol-hMGL form.

The 2D ^1^H-^15^N HSQC spectra of sol-hMGL and of all mutants demonstrated good chemical shift dispersion, confirming a presence of well-defined secondary structures, although a rather significant number of resonances were inhomogeneously broadened and poorly resolved. The observed broadening originates from the contribution of slow to intermediate chemical exchange rate on the NMR time scale between multiple conformational states of hMGL. As a consequence, spectra contain less than the expected number of resonances, thus, preventing successful sequential NMR resonance assignment. In the case of W289L (Fig. 3c) and L232G (data not shown) mutants severe line broadening indicates that these substitutions provide dramatic effect on the stability of tertiary structure and conformational sampling of enzyme.

The robustness of proteins is the ability of the protein sequence to adopt global structural changes induced by mutational perturbations without suffering loss of function^38^. From the data presented here, it is evident that either conservative or nonconservative substitution at position 289 results in global conformational changes in hMGL. Our results show that only a conservative substitution is tolerated by hMGL. The nonconservative substitutions at position 289 and 232 were not tolerated highlighting the functional importance of interactions between Trp-289 and Leu-232.

### Ligand Accessibility to the Active Site of hMGL Mutants

hMGL is characterized by the presence of an extensive flexible lid domain comprising residues 151–225, which form three helices α4, α5 and α6 as well as several surface loops. The lid covers and regulates access to the binding site of the enzyme. In the open form, the active site is exposed to the solvent. Monitoring chemical shift perturbations (CSPs) in the downfield region of ^1^H NMR spectra can unambiguously identify ligand penetration into the binding pocket. These CSPs are indicative of specific binding and the corresponding conformational changes. When there is a mutationally-induced irreversible shift toward the closed conformation or a conformation with restricted entrance to the binding pocket of hMGL, the lid obstructs the entrance to the active site, preventing ligand penetration into the binding pocket and resulting in no observable CSPs. However, when there is even a small amount of open form in a pre-existing equilibrium with a dominant closed form, complexation with a high affinity ligand can shift the equilibrium towards the holo-hMGL complex, which is clearly detectable by the presence of CSPs in the downfield region of ^1^H NMR spectra. Thus, observation of CSPs upon binding hMGL constructs to known high affinity ligands can provide important information regardless of the conformational equilibrium and ligand accessibility of conformers to the active site.

**Figure 6:**
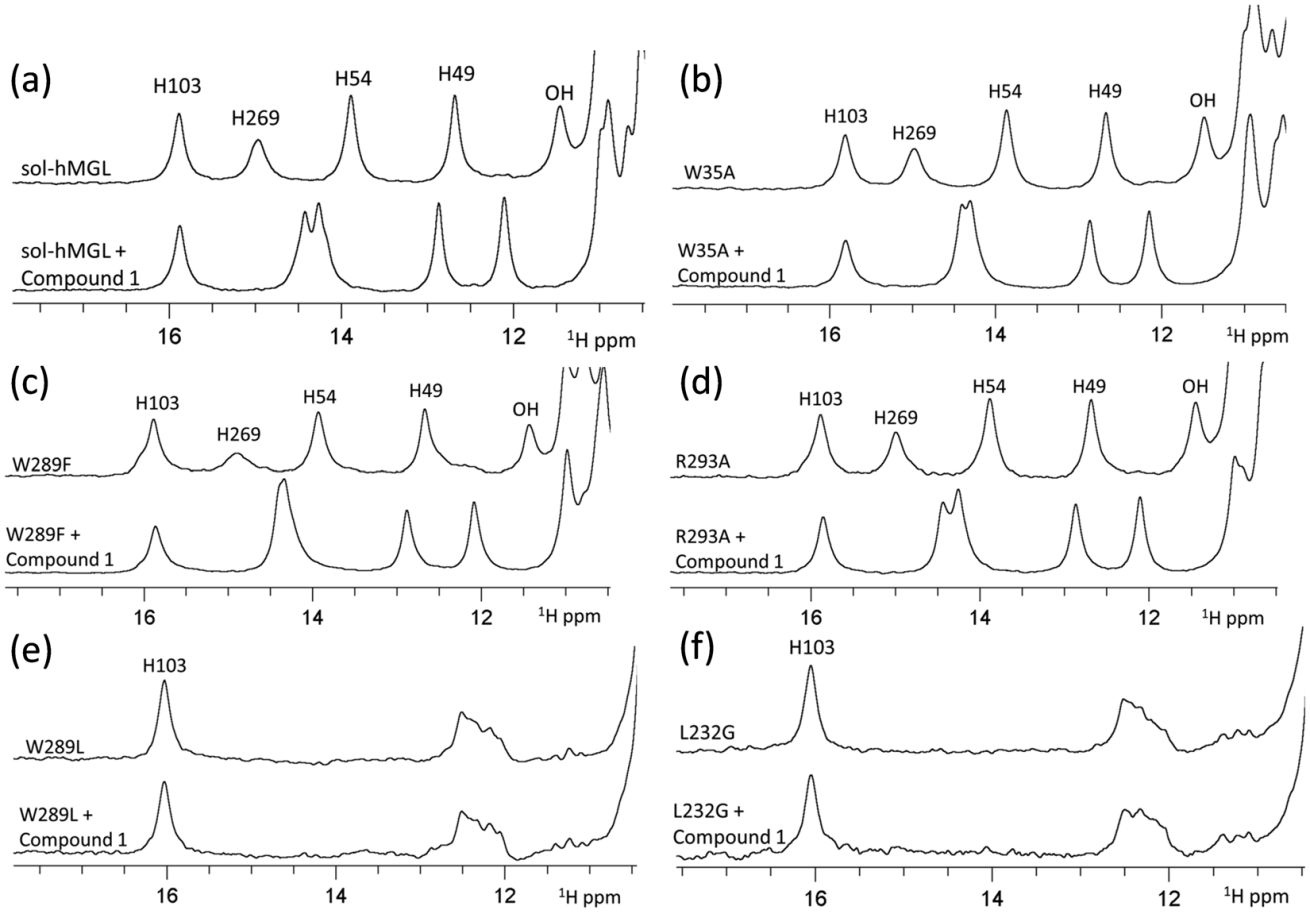
Effect of hMGL binding with Compound-1 on the downfield resonances of (**a**) sol-hMGL; (**b**) W35A; (**c**) W289F; (**d**) R293A; (**e**) W289L and (**f**) L232G at pH 7.4, T = 310 K.

Compound-1, 2-cyclohexyl-6-{[3-(4- pyrimidin-2-ylpiperazin-1-yl)azetidin-1-yl]carbonyl}-1,3-benzoxazole has high affinity (*K*_*i*_ ~10 nM) and selectivity for hMGL^22,39^. We have used this potent and reversible inhibitor (as AM10212) to evaluate ligand accessibility to the active site of hMGL mutants. Figure 6a shows the characteristic downfield ^1^H NMR pattern of sol-hMGL. Formation of a reversible complex between sol-hMGL and compound-1 caused significant perturbations of His-269, His-54, His-49 and unassigned OH resonances. As a result, a new specific NMR pattern indicating full occupancy of the hMGL binding site by compound-1 was observed. The downfield NMR spectral patterns were compared for free forms and complexes of compound-1 with W35A (Fig. 6b), W289F (Fig. 6c) and R293A (Fig. 6d) mutants. These data provide evidence for binding site occupation in these mutants and the formation of complexes similar to the complex observed for sol-hMGL with compound-1. In contrast, the ^1^H NMR patterns of W289L (Fig. 6e) and L232G (Fig. 6f) mutants consist of only two groups of peaks, presumably due to the significant broadening of some downfield resonances beyond detection. Addition of excess inhibitor does not induce any detectable changes in the NMR spectra of either mutant, supporting the notion of a drastic reduction in access to the binding pocket due to the conformational shift toward very dynamic conformational states. Thus, our NMR binding experiments serve as an additional line of evidence for a conformational shift towards ligand-inaccessible dynamic forms triggered by distal single point mutations at 289 and 232 positions.

### HDX-MS Analyses of wild type hMGL and W289L Mutant

**Figure 7:**
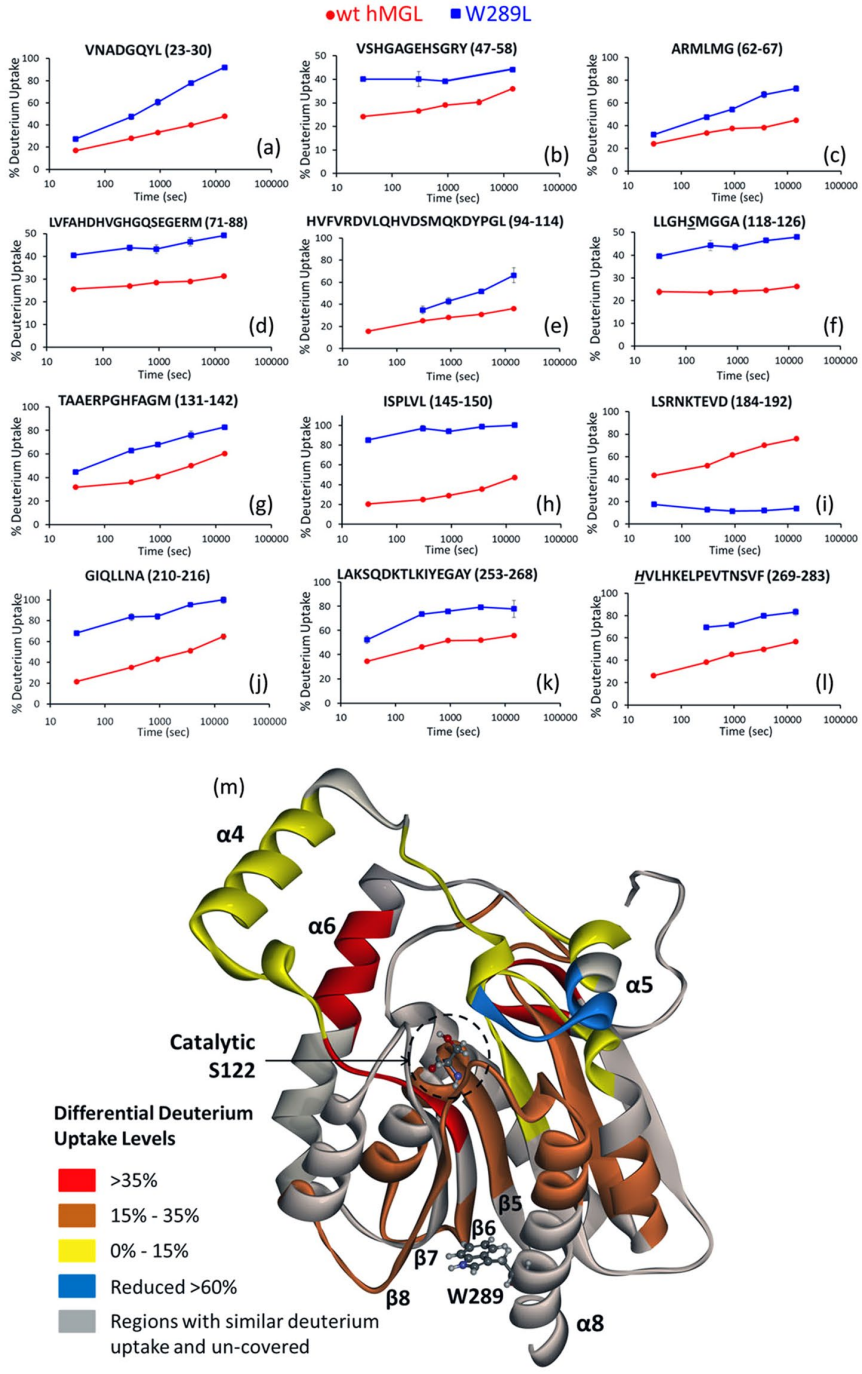
Plots of deuterium uptake vs time (30 s, 5 min, 15 min, 1 h and 4 h) for the 12 peptides (**a–l**) showing statistically different behavior for W289L mutant as compared to wt hMGL. Uncertainties (1σ), indicated by bars, reside inside the plotted symbol. (m) Differential deuterium uptake profiles of wt-hMGL and W289L at 4 h mapped onto the crystal structure of hMGL (PDB:3HJU). Increased differential deuterium uptakes are color-coded from yellow to red and reduced uptake is colored blue. Grey indicates regions where the deuterium uptake levels in the wt hMGL and W289L mutant are statistically the same. Grey, in addition, denotes regions where peptic peptides were not observed.

It was not feasible to obtain dynamic data for hMGL mutants based on the NMR measurements of relaxation parameters due to the substantial NMR line broadening. Therefore, we have used current state-of-the-art HDX-MS methodology. Wild type hMGL and the W289L mutant were analyzed by HDX-MS to probe the effect of nonconservative replacement of Trp-289 on the conformation and dynamics of the enzyme and to correlate dynamic changes with the loss of function. HDX-MS experiments, which measure the H/D ratios among backbone amide hydrogens of the amino acid sequence, provide the ability to compare the combined effects of hydrogen-bonding of amide groups and solvent accessibility between hMGL wild type and mutant. Peptic peptides of hMGL species were identified from MS/MS spectra, and the peptides are plotted as bars against the respective protein sequences in Fig. S5. Online pepsin digestion of deuterium labeled, quenched samples of both hMGL enzymes resulted in a total of 101 peptides for wild type hMGL (Supplementary Fig. S5a) and 43 peptides for W289L (Supplementary Fig. S5b). The set of 43 peptides observed in the mutant are also observed in wild type hMGL. This set of peptides covers 75% of the hMGL sequence with some redundancy. This redundant set can be reduced to a subset of fifteen peptides that achieves maximum sequence coverage while avoiding residue redundancy. This subset enables head-to-head comparisons of deuterium uptake by wild type hMGL and W289L peptides, thus providing insight into the changes in dynamics induced by mutation. Table 2 tabulates the deuterium uptake as a function of time for peptides of the wild type hMGL and mutant. Peptides showing deuterium uptake greater than 10% are shown in Fig. 7.

**Table 2:** Percentage deuterium uptake levels for the peptides detected in both wt hMGL and W289L for D_2_O immersion of 30 s to 4 h.

**Peptide**	**Sequence**	**30 sec**		**5 min**		**15 min**		**1 hours**		**4 hours**	
		**wt hMGL**	**W289L**	**wt hMGL**	**W289L**	**wt hMGL**	**W289L**	**wt hMGL**	**W289L**	**wt hMGL**	**W289L**
VNADGQYL	23–30	17 ± 0.4	27.4 ± 1.5	28 ± 0.6	47.5 ± 2.6	33.3 ± 0.9	60.5 ± 3	39.9 ± 0.7	77.8 ± 0.7	47.8 ± 0.9	91.9 ± 3
VSHGAGEHSGRY	47–58	24.2 ± 0.8	40.1 ± 0.8	26.6 ± 0.8	40.1 ± 3.2	29.1 ± 1	39.2 ± 1	30.3 ± 1.2	—	36 ± 0.3	44.1 ± 4.6
ARMLMG	62–67	24 ± 0.6	32.1 ± 0.7	33.6 ± 0.3	47.5 ± 1.4	37.5 ± 0.6	54.2 ± 2.5	38.3 ± 0.6	67.3 ± 2.8	44.8 ± 0.8	72.6 ± 2.6
LVFAHDHVGHGQSEGERM	71–88	25.6 ± 0.3	40.5 ± 0.2	27 ± 0.1	43.8 ± 1.3	28.5 ± 0.3	43.2 ± 1.9	29 ± 0.6	46.4 ± 1.9	31.3 ± 0.7	49.2 ± 0.3
LLGHSMGGA	118–126	23.9 ± 1.3	39.6 ± 0.8	23.6 ± 0.3	44.3 ± 2.3	24.1 ± 0.8	43.6 ± 1.7	24.6 ± 0.1	46.5 ± 1.2	26.3 ± 0.7	48 ± 0.5
TAAERPGHFAGM	131–142	31.9 ± 0.1	44.8 ± 0.5	35.9 ± 0.2	63 ± 2.2	40.9 ± 1	67.9 ± 1	50 ± 0.9	76.1 ± 3.4	60.4 ± 0.8	82.8 ± 1
ISPLVL	145–150	20.6 ± 0.4	85.2 ± 1	25 ± 0.1	96.8 ± 2.9	29.1 ± 0.7	93.9 ± 1.8	35.5 ± 1.8	98.5 ± 2.4	47.3 ± 0.9	100.2 ± 0.1
ANPESATTF	151–159	84.2 ± 0.3	91.8 ± 2.1	100.5 ± 0.9	99.3 ± 4.3	101 ± 0.4	93.9 ± 2.2	95.4 ± 2.1	96.4 ± 6.2	93.8 ± 1.2	98.7 ± 1.8
KVLAAKVLNL	160–169	32.7 ± 3.4	32.3 ± 2.1	—	88.4 ± 4	73.6 ± 0.1	96.5 ± 3	87.8 ± 2.8	—	90.3 ± 1.4	100.3 ± 2.6
SLGPIDSSVL	175–184	61.9 ± 0.6	94.2 ± 0.4	77.4 ± 0.9	93.6 ± 4.3	83.8 ± 0.8	91.3 ± 4.3	85.8 ± 1.4	95.6 ± 8.6	89.1 ± 1	100.1 ± 1.3
LSRNKTEVD	184–192	43.3 ± 0.6	17.5 ± 0.6	52.1 ± 0.3	13 ± 1.3	61.5 ± 0.8	11.6 ± 1	70.2 ± 1.4	12.1 ± 1.1	76 ± 1.3	14 ± 1.1
YNSDPLIC	194–201	29.8 ± 0.5	89.9 ± 3	50.5 ± 1	89.6 ± 0.1	60.9 ± 0.8	80.4 ± 0	69.8 ± 2.4	77.4 ± 0	76.2 ± 2.3	77.4 ± 0
GIQLLNA	210–216	21.3 ± 0.6	68.1 ± 0.9	35.2 ± 0.3	83.6 ± 3.3	43.1 ± 1	84.2 ± 2.7	51.1 ± 2.2	95.5 ± 1.5	65 ± 2.6	100.8 ± 3.3
LAKSQDKTLKIYEGAY	253–268	34.5 ± 0.6	52.3 ± 3.1	46.3 ± 1.2	73.5 ± 0	51.6 ± 0.4	75.8 ± 1.5	52 ± 1.7	79.2 ± 0	55.9 ± 1.3	77.8 ± 7.1
HVLHKELPEVTNSVF	269–283	26.2 ± 0.2	—	38.3 ± 0.1	69.5 ± 1.8	45.2 ± 0.3	71.7 ± 1.6	49.8 ± 0.9	79.8 ± 1.9	56.6 ± 1	83.2 ± 3.2

The data points are presented as mean ± standard error (1σ), which are computed from data observed in three replicate experiments. The values are adjusted for back exchange.

The deuterium uptake data reveal that 94% of reporting amides affected by substitution of Trp-289 with Leu exhibit significantly higher deuterium uptake rates over time (Fig. 7). An exception is observed in one peptide 184–192 that exhibits a significantly decreased deuterium uptake rate (Fig. 7i). The overall deuterium uptake rate increase suggests that hMGL becomes more dynamic in a number of distinct regions upon nonconservative mutation at position 289. Specifically, the data for peptide 118–126, containing the catalytic Ser-122 residue (Fig. 7f), indicate that this region of wild type hMGL is highly protected, resulting in small rates for deuterium exchange. This is consistent with a pronounced conformational rigidity of the hMGL nucleophilic elbow (sharp turn between strand β5 and helix α3) where the catalytic Ser-122 resides. However, upon mutation this region demonstrates 22% greater deuteration after the first 30 s and a more rapid subsequent deuterium uptake rate, indicating that this region in W289L is less protected. Similarly, peptide 269–283, which contains the catalytic triad His-269, is only partially protected and shows a substantial stepwise increase (22%) in deuterium uptake in W289L (Fig. 7l). In this case mutation likely perturbs only part of this peptide, residing in the loop located between strand β8 and helix α8 (269–275), due to rapidly exchanging amide hydrogens. It seems reasonable to assume that this region of the enzyme undergoes substantial conformational change in concert with the region 118–126, which contains the catalytic Ser-122. This behavior is indicative of conformational changes within the active site of the enzyme.

HDX-MS data from peptide 47–58 report the loop region between helix α1 and β4 and part of strand β4. Gly-50 and Ala-51 in this loop are critical oxyanion hole-forming residues^21^. For this region the deuterium uptake jumps >10% for all exchange times (Fig. 7b). Additionally, peptide 118–126, which shows greater deuterium uptake in the mutant, also reports on the region containing Met-123 and Gly-124. These residues are involved in the formation of the hMGL oxyanion hole. Therefore, the substitution of Trp289 with a non-aromatic residue induces the oxyanion hole to be more dynamic and less preorganized.

Remarkably, the highest difference in the degree of deuterium incorporation in the wild type hMGL and mutant (≈20% vs. ≈80% after 30 s exchange) was detected for the peptide 145–150 (Fig. 7h), which corresponds to a loop between strand β5 and helix α4 that characterizes the beginning of the lid. In fact, this region connects the α/β-hydrolase core domain of hMGL with the lid domain (Fig. 1) and may have direct effects on helix α4. It is important to recall that open-closed transitions of the lid involve the rolling motion of helix α4 over the active site^21^. Therefore, this observation upon substitution of Trp-289 suggests a structural change that may affect substrate access to the active site, compromising enzymatic activity.

Additionally, two peptides derived from the lid domain (184–192) and (210–216) showed significant changes in deuterium incorporation in the mutant. Peptide 184–192 (Fig. 7i), which samples part of the loop and helix α5 in the lid domain, is the only region demonstrating a drastic reduction in the deuterium incorporation in W289L. In W289L peptide 184–192 (Fig. 7i) is relatively inert at all exchange times, suggesting that this part of the lid likely becomes partially buried due to the movement away from water into the solvent-shielded hydrophobic core of enzyme. Peptide 210–216 (Fig. 7j) reports on part of helix α6 in the lid domain, which becomes more dynamic and available to exchange with solvent in W289L. Collectively, these data strongly suggest substantial structural rearrangements within the loop that connects the hMGL core with the lid domain as well as the entire lid domain in mutant.

We have identified several other peptides from different regions of enzyme that display significant increases in the deuterium uptake for the W289L mutant compared to the wild type (Fig. 7a,c–e,g,k). These peptides provide additional evidence for the global changes in hMGL structure and dynamics upon nonconservative substitution at the position 289.

Regional differential deuterium uptake profiles calculated as a difference between the percent deuterium uptake in wild type hMGL and W289L mutant at 4 hours exchange duration were color-coded onto the enzyme’s wild type X-ray crystal structure (Fig. 7m). The regions of hMGL where the peptic peptides were not observed are shown in grey. In addition, regions where the deuterium uptake was not affected by mutation are also colored in grey. Overall no significant changes in H/D rates of exchange were detected in 55% (with respect to wild type sequence coverage) of the enzyme.

In summary, our HDX-MS profile of hMGL reveals significant dynamic differences in distinct regions between wild type and W289L mutant, indicating a major conformational shift upon mutation. The W289L construct becomes more dynamic in regions key to the enzymatic function including the catalytic triad, oxyanion hole, and lid domain. Thus, the mutant undergoes greater conformational sampling and consequently greater exchange with bulk solvent. The conformational shift to less rigid structures with compromised active site preorganization is a likely reason for the observed loss of hMGL function.

### Nanosecond MD simulations for wild type hMGL and W289L mutant

The aim of MD simulations in this study was to capture the effect of a distal single point mutation on the open state of hMGL. The X-ray structure of wild type hMGL (PDB: 3HJU) was used as a template for defining a starting model for MD simulations of wild type and the W289L mutant. The W289L mutation was introduced into the model using the graphical user interface Maestro from Schrodinger software. MD simulations were carried out for 200 ns for each enzyme model. Figure S6a shows a plot of the C_α_ atoms root-mean-square deviation (RMSD) evolution. Compared to the initial structure, the open conformation equilibrated at ~100 ns with an RMSD value of 1.58 ± 0.12 Å. W289L also equilibrated at ~100 ns but with an RMSD value of 2.18 ± 0.15 Å, demonstrating that the mutant adopts a different conformational state than the wild type hMGL. The thermodynamic stabilities of these two enzymes were estimated by their root-mean-square fluctuation (RMSF) values based on C_α_ atom fluctuations for each residue around its average position. Figure S6b represents the RMSF value per residue for wild type hMGL and W289L during a 200 ns MD simulation. The lid domain (residues 151–225) is the most dynamic part of hMGL, demonstrating substantial conformational changes (Supplementary Fig. S7) and altered flexibility (Supplementary Fig. S8) upon mutation. Nearly all other residues outside the lid show a slight increase in flexibility, indicating a subtle global effect. Overall, the W289L mutant has a larger conformational flexibility compared to the wild type (Supplementary Fig. S6b and S9).

**Figure 8:**
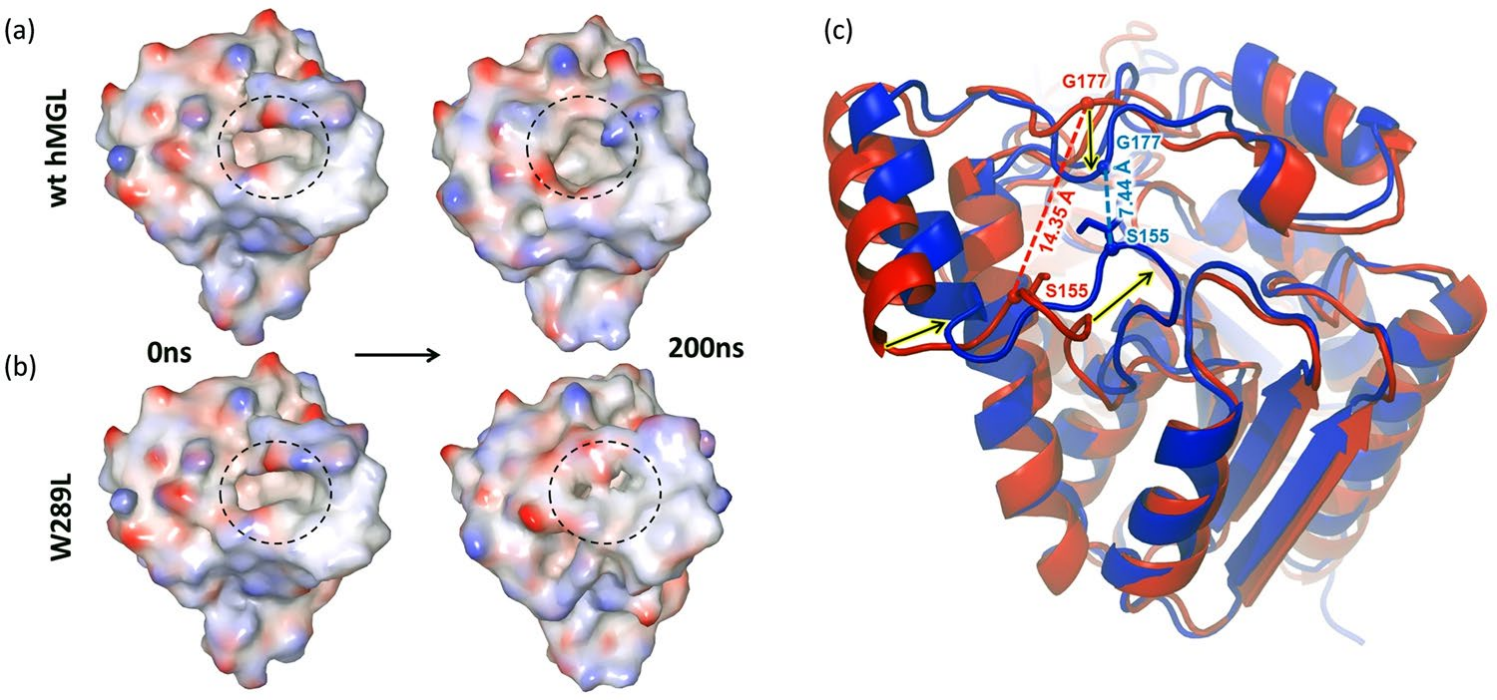
MD simulation results for wt hMGL and W289L mutant. Black dashed circles mark the substrate entrance area into the hMGL active site (binding pocket). (**a**) The wild type retains the open conformation throughput the 200 ns simulation. (**b**) W289L mutant moves towards the restricted conformation after 100 ns. Surface colors illustrate the electrostatic potential with red representing negative charges and blue representing positive charges. (**c**) Overlapping of W289L three-dimensional structures at 0 ns (red) and after 200 ns (blue) of MD simulation study. The C_α_ distance between the two representative residues Ser-155 and Gly-177 decreased 2-fold. The black arrows indicate the direction of the helix/loop movement.

The C_α_ atoms distance between the lid domain residues Phe-159 (helix α4) and Gly-210 (helix α6) may be used as a sensitive probe to analyze access to the hMGL active site^40^. The 14.7 Å distance is indicative of a fully open conformation, whereas a distance of 7.8 Å is indicative of a closed conformation. Thus, two extreme conformational states of hMGL have almost a 7 Å difference in the distance between Phe159 and Gly210. Figure S10 shows the Phe159-Gly210 distance for our two simulated systems. The difference in this distance between wild type and W289 mutant is 2.5 Å. This suggests that the access to the catalytic cleft in our case is only partially occluded, restricting binding of the substrate. Changes to the surface and entrance to the catalytic site of hMGL during MD simulations are shown in Fig. 8a,b. The entrance to the active site remained open throughout the 200 ns simulation for the wild type enzyme. However, tightening of the entrance was already observed at 100 ns for the W289L mutant. The ribbon superposition of W289L structures before and after the MD simulation (Fig. 8c) showed substantial structural rearrangements within two loops from the lid domain, one connecting β6 with α4 (residues 151–156), and another loop connecting α4 with α5 (residues 176–178). The inward movement of helix α4 coupled with tightening of both loops surrounding it, may hinder or prevent the substrate from binding to the active site. In summary, our MD simulations clearly show that the lid region of hMGL is substantially involved in the conformational changes, restricting access to the catalytic site of enzyme upon substitution of Trp-289 with Leu.

## Discussion

It is well known that proteins have the ability to maintain their function, whilst tolerating environmental changes and mutations^41^. Proteins evolved toward a robust design^42^, and they can differ substantially in their robustness to mutations. However, this robustness is associated with sensitivity to perturbations at certain crucial sites^43^. In many cases, proteins may respond drastically to mutations that occur at sites far away from the active site and/or binding pocket^44^. Currently, communication between distant sites is recognized as one of the fundamental characteristics of enzyme function and facilitates signal transmissions from one functional site to another remote site through a number of pathways during the course of biological function. These long-range interactions are extraordinary considering that many other residues, closer or even adjacent to the active site residues, do not affect protein function. Furthermore, proteins can adopt a variety of open and closed conformational states depending on environmental modulators, ligand binding, and mutagenesis. Knowledge of how remote substitutions can redistribute protein conformational states of hMGL is critical for discovery of selective and potent allosteric inhibitors for this important pharmaceutical target.

In the present work we have found that substitution of Trp with Leu at position 289 drastically decreases the catalytic efficiency of hMGL despite the fact that it is more than 18 Å away from the catalytic triad (Fig. 1). This substitution caused substantial CSP of hydrogen bonded histidine side chain resonances and, consequently, downfield ^1^H NMR patterns (Fig. 4), indicating changes in the chemical environment far from the Trp-289 residue. These changes occur at distinct, functionally important regions of the enzyme’s structure, such as the catalytic triad (His-269), oxyanion hole (His-49) and lid domain (His-54). In addition, the 2D NMR ^1^H-^15^N HSQC spectrum of W289L (Fig. 5b,d) supports the findings from 1D NMR spectra, which have unambiguously demonstrated the global conformational changes induced by local perturbation at position 289. Analysis of NMR data suggests that the equilibrium for W289L is shifted towards an ensemble of very dynamic conformers.

In addition, NMR binding data (Fig. 6e) demonstrated ligand-inaccessibility of W289L conformers, presumably due to the dramatic fluctuations of tertiary structure. In accord with NMR data, HDX-MS analysis independently evidenced the presence of a major conformational transition and consequent significant changes in the conformational dynamics of functionally important regions triggered by the W289L mutation. Finally, MD simulations for this mutant revealed an increased overall conformational flexibility and restricted accessibility to the binding pocket with respect to the wild type. Computational results also highlighted substantial involvement of the lid domain in this conformational transition of hMGL. Thus, our study shows remarkable agreement between different biophysical techniques as well as the integrity of experimental and computational results.

With the aim of identifying communication motifs, the residues proximal to Trp-289 in space (Fig. 1) were examined as potential hot spots. Notably, the same conformational shift towards very dynamic conformers as for W289L and dramatic loss of activity were also observed in the case of the nonconservative substitution of Leu-232, which is proximal to Trp-289 in space but distal in sequence. The finding of two residues proximal in the folded structure that have deleterious effects upon nonconservative replacement, clearly evidence the involvement of Trp-289 and Leu-232 in the network of energetically coupled residues and indicates their critical role in the conformational transitions of hMGL. In contrast, nonconservative replacement of another neighboring residue Arg-293, which is proximal to W289 in space and sequence, does not alter hMGL conformational equilibrium and catalytic efficiency, demonstrating a high level of tolerance to this substitution. The same high level of tolerance was also found in the case of W289F conservative mutation, showing that the subtle replacement of tryptophan with the smaller aromatic side chain of phenylalanine maintains the aromatic interactions with Leu-232 necessary for activity. Overall, experimental mutagenesis, kinetics and NMR data pointed out that Trp-289 and Leu-232 nonconservative mutations dramatically affect 2-AG and the inhibitor binding capability of hMGL. Importantly, both residues are not in direct contact with the binding pocket nor with the catalytic triad residues that are over 18 Å away. Chemical shift perturbations in 1D and 2D NMR spectra of both W289L and L232G mutants directly indicate that local perturbations at the 289/232 sites are propagated to the distal, functionally important sites of hMGL, which include the catalytic triad and the lid domain, via pre-existing intramolecular communication pathways in the native enzyme. This allosteric propagation causes different hMGL sites to communicate with each other and shifts the relative distribution of conformations within the pre-existing ensemble toward less-ordered, ligand-inaccessible forms. It should be noted that only highly connected residues in the communication networks, referred to as hubs, have the potential to allow a proper and effective flux of information to the distal sites throughout the enzyme’s 3D structure. Moreover, hubs can also play a critical role in structural stability and function of proteins^45,46^.

Based on multiple lines of experimental evidence, including NMR and HDX-MS, we suggest that the nonconservative W289L and L232G mutations impair allosteric communications between regulatory sites, due to the fact that these residues act as hubs allowing distal communication to functional sites of hMGL, rather than just a destabilization of the protein fold. Although 1D and 2D NMR structural assessment revealed major structural and dynamical changes upon these mutations, both mutants maintain a similar α/β- hydrolase fold as the wild type protein. In accord with NMR data, HDX-MS results also suggested that the W289L mutant is also folded, as we did not observe high levels of deuterium incorporation in most regions of the enzyme. However, the high levels of deuterium uptake in key regions shown in Fig. 7 indicate a significant effect on conformation and dynamics at the active site and in certain other distinct regions distal from the catalytic center. This evidence strongly suggests that the overall flexibility of the enzyme was increased upon substitution of these residues. In addition, MD simulations data also indicate that W289L exhibits a larger conformational flexibility as compared to the wild type. Thus, nonconservative replacements at both 289 and 232 positions significantly disrupt the dynamic infrastructure and modulate long-range allosteric signaling that propagates to functionally important regions. At the same time, our NMR and HDX-MS data suggest that the identified loss-of-function distal mutations may also induce substantial changes in the tertiary structure of hMGL, leading to the molten globule state. Therefore, Trp-289 and Leu-232 are likely to be not only critical residues for long-range communication but also essential for the functional hMGL tertiary structure.

These residues as hubs may play an important role in the integration of β7 and α8 secondary structural elements in the 3D structure of hMGL. It appears that the spatial proximity and essential contacts between these two hydrophobic residues create steric hindrance and likely control the geometry of contacts between strand β7 and helix α8. Therefore, nonconservative substitution of Trp-289 relieves the steric hindrance imposed by the indole ring, causing major changes in the native communication pathways required for hMGL function and shifting the conformational equilibrium toward the inactive states. This conformational shift is irreversible in W289L, according to our NMR results, and communications are redirected toward other regions compared to native hMGL, as shown by HDX-MS data and MD computations. The same is applicable for L232G substitution. Hence, Trp-289 and Leu-232 are strong hubs for long-range communication between distal sites and the hMGL active site. These residues play an essential role in structural integrity and function of hMGL.

There are three mechanisms invoked to explain the transmitted effects of distal mutations causing a change in functional properties^44^. The first mechanism implies that the delicate equilibria between conformers can be disturbed by a single mutation due to alteration of intra/inter-molecular contacts that stabilize particular conformations. A second one infers that changes within a side chain network result in a different pattern of molecular interactions. These changes, in turn, will be propagated due to a second shell of amino acids that have altered interactions with their surroundings. A third mechanism implicates only changes in protein conformational motion. Even though mutant and wild type structures may be identical, the functional effect of the mutation is to alter mobility. The overall effects may be a combination of these mechanisms. Our current experimental and computational evidence strongly support the conclusion that the effect of a remote mutation in hMGL is transmitted through the enzyme’s structure by a combination of these mechanisms. A disturbance of the pre-existing delicate equilibrium in hMGL due to alteration of important intra-molecular interactions is the dominating mechanism causing a loss of function for this enzyme. Our results also support the notion that a change in the side chain network of interactions may be a second important mechanism. Only a subtle contribution was found from the third mechanism involving protein conformational motions. Thus, in the case of hMGL, a major conformational change is required in combination with minor local changes for the transmission of distal mutational effects to the active site.

In summary, we have found that mutations at a location over 18 Å away from the active site of hMGL highly influence the catalytic triad, implying a critical role of this remote site in the allosteric regulation of enzyme catalysis. We have defined remarkable changes in communication between this site and the hMGL active site upon mutation. We have identified Trp-289 and Leu-232 as key residues that act as hubs in the communication flow through the enzyme. Our results provide insight into a mechanism by which a single distal point mutation has a major impact on hMGL activity by modifying its structure, conformational dynamics, and allosteric communications. This is the first reported allosteric site in an important pharmaceutical target – hMGL. This discovery provides exclusive opportunities for development of new strategies toward modulating hMGL function and identification of novel pharmaceuticals.

## Methods

### Site-directed Mutagenesis, Expression and Purification

The N-terminal His-tagged human MGL enzyme with L169S/L176S substitutions was used as a template for site directed mutagenesis. Six different point mutations were introduced in this sol-hMGL template, namely W35A, W289L, W289A, W289F, L232G and R293A. The full DNA sequence for the stated mutations was submitted to GenScript (Piscataway, NJ). Synthesized DNA were provided upon full sequencing and cloning in pET-45b(+). The mutated plasmids were then transformed and expressed in BL21 (DE3) *E. coli* cells. The expression and purification of each recombinant protein was performed as previously prescribed^26,28^. ^15^N-labeled samples of sol-hMGL were produced in phosphate minimal media supplied with ^15^N NH_4_Cl (Cambridge Isotope Labs)^26^. The open sol-hMGL conformer undergoes spontaneous transition to the closed conformation upon prolonged incubations in aqueous phosphate buffer. For the mutants W289L, W289F and L232G, uniformly ^15^N-labeled cells were grown in Spectra-9 media for bacterial cell growth (Cambridge Isotopes Labs) and purified.

### Assay for MGL Enzymatic Activity

The hydrolysis activity of hMGL was assessed by incubating the purified enzyme with endogenous substrate and quantifying the product arachidonic acid (AA) with a high-performance liquid chromatography (HPLC). The substrate 2-AG was generously supplied by NIH (Bethesda, MD), while AA was purchased from Nu-Chek Prep (Elysian, MN). 2-AG was dispersed in 280 µL reaction buffer (50 mM Tris-HCl, 5 mM MgCl_2_, 1 mM EDTA) containing 0.1% BSA. The final elution, containing between 20 ng to 2 µg total proteins in a volume of 5 µL, was added to start the reaction, and the solution was allowed to react for 20 min at 37 °C. To assure maximum reaction rates, the concentration of 2-AG ranged between 13 µM and 400 µM. The reaction was quenched by adding 50 µL reaction aliquot to 200 µl acetonitrile. The mixture was centrifuged and injected onto the HPLC column (4.6 × 50 mm Zorbax XDB-C18, Agilent Technologies, CA). 2-AG and AA were separated at a flow rate of 1 mL/min. The mobile phase consisted of solvent A (acetonitrile) and solvent B (acetonitrile/water/o-phosphoric acid, 54:40:6 v/v/v). The gradient used was from 5% to 100% solvent B in 8 minutes. To measure the amount of substrate conversion, the released arachidonic acid was analyzed by HPLC with UV detection at 204 nm. The velocity data generated from UV-Vis and HPLC results were fitted to a Michaelis-Menten plot using nonlinear regression in GraphPad Prism 5.0 (San Diego, CA) with the aim to estimate *V*_max_, *K*_m_, *k*_cat_ and catalytic efficiency (*k*_cat_/*K*_m_).

### Circular dichroism spectroscopy

Circular dichroism measurements were performed on a Jasco J-815 CD spectrometer with a Peltier temperature controller and single cuvette holder. Conformational changes in the secondary structure of protein were monitored in the far-UV region between 190 to 260 nm with a protein concentration of 10 µM (300 µL) in a quartz cuvette with a path length of 1 mm. The solution conditions were 20 mM sodium phosphate, 100 mM NaCl, 1 mM DTT buffer, pH 7.4.Three accumulations of scan were taken (within 600 HT voltage range) and averaged to get the complete spectra.

### Nuclear Magnetic Resonance Spectroscopy and Data Analysis

All NMR experiments were performed on a Bruker AVANCE II 700 MHz spectrometer. Protein samples were prepared in 95% H_2_O, 5% D_2_O buffer containing 20 mM sodium phosphate, 200 mM NaCl and 1 mM DTT at pH 7.4. Sample volumes were between 0.5 mL to 0.6 mL. Chemical shifts were referenced to sodium 2,2-dimethyl-2-silapentane-5-sulfonate (20 µM final concentration), which served as an internal standard. All 1D ^1^H NMR experiments were recorded using a Watergate pulse sequence (p3919fpgp) for water suppression. Two-dimensional ^1^H-^15^N HSQC NMR spectra were recorded using the standard pulse sequences supplied with AVANCE 700 spectrometer, as detailed before^26^. The data were processed and visualized using Topspin 3.2 (Bruker). For ligand binding NMR experiments Compound-1^47^ was synthesized at the Center for Drug Discovery. A 50 mM stock solution of compound-1 in DMSO-*d*_6_ was used to achieve a 2-fold molar excess of ligand to hMGL.

### Mass Spectrometry of wild type hMGL and W289L hMGL Mutant

Peptic peptides were generated by passing 17 pmol of protein through an Enzymate pepsin column (Waters, Milford, MA) and identified from tandem MS (MS/MS) data observed on a Thermo LTQ Orbitrap Elite (Thermo Fisher, San Jose, CA). On the most abundant precursor ions, one full mass spectral acquisition triggered six scans of MS/MS with activation by collision-induced dissociation (CID). Peptides were identified by the MASCOT (Matrix Science, Oxford, UK) database search engine with the following parameters: enzyme, none; oxidation (M) as a variable modification; MS tolerance, 20 ppm; MS/MS tolerance, 0.6 Da; peptide charge of +2, +3, and +4.

### HDX-MS and HDX Data Processing

For HDX-MS analyses, the wt hMGL and W289L hMGL mutant protein stock solutions were diluted in H_2_O buffer (20 mmol/L sodium phosphate, 150 mmol/L sodium chloride, 2 mmol/L TCEP at pH 7.4) to prepare a 5 µmol/L final concentration and equilibrated at 1 °C. HDX was conducted on a HDX PAL robot (LEAP Technologies, Carrboro, NC). Protein solutions (5 µL) were diluted into 25 µL D_2_O buffer (20 mmol/L sodium phosphate, 150 mmol/L sodium chloride, 2 mmol/L TCEP at pD 7.4) at 25 °C. At selected times (0 s, 30 s, 5 min, 15 min, 1 h, and 4 h) the HDX sample was quenched by mixing with 35 µL quench buffer (3 mol/L urea, 0.1 mol/L sodium phosphate at pH 2.5) at 1 °C. The quenched solution was injected into an on-line immobilized pepsin column for 3 min. The digested protein solution was trapped on a C18 guard column (1.0 mm diameter x 5 mm length, 5 µm; Grace Discovery Sciences, Deerfield, IL) and separated with a C18 analytical column (1.0 mm diameter x 5 mm length, 1.9 µm, Hypersil GOLD, Thermo Scientific) via a Thermo Fisher Ultimate 3000 UPLC with a 9.5 min gradient operated with a binary mixture of solvents A and B at 50 µL/min flow rate. The gradient settings used were: 5% to 35% solvent B for 3 min, 35% to 60% solvent B for 5 min, 60% to 100% solvent B for 0.5 min, isocratic flow at 100% solvent B for 0.5 min, and a return in 5% solvent B for 0.5 min. Solvent A was water containing 0.1% formic acid and solvent B was 80% acetonitrile and 20% water containing 0.1% formic acid. LC connection lines and valves were housed in a refrigerated compartment at 2 °C. Peptides were mass analyzed on a Thermo Orbitrap Elite. The instrument settings were: spray voltage, 3.7 kV; sheath gas flow rate, 25 (arbitrary units); capillary temperature, 275 °C. In the Orbitrap stage, MS spectra were acquired with the resolution set at 60000. Three replicates for each ion-exchange time point were obtained. From mass spectra obtained during HDX-MS experiments, the centroid of each deuterated peptide envelope and the relative deuterium uptake by each peptide were calculated by HDX WorkBench^48^. Corrections for back exchange were made by considering the values of 80% deuterium content of the exchange buffer and an estimated 70% deuterium recovery. Paired t-tests were used to verify deuterium uptake differences.

### Molecular Dynamics Simulations

Desmond^18,49^ program in Schrodinger 2015–3 with OPLS3 force field^50^ was applied to build an aqueous biological system, followed by MD simulation. The SPC model was used to simulate water molecules^51,52^. The orthorhombic periodic boundary conditions (10 × 10 × 10 Å^3^) were set up to specify the shape and size of the repeating unit. The minimum number of sodium and chloride ions was used to neutralize the system and they were placed randomly in the solvated system. Salt (sodium and chloride) with a concentration of 0.15 M was also added to the system. An option of Relax Model System Before Simulation was selected. A series of minimizations and short molecular dynamics simulations were performed to relax the model system before performing the final simulation. Lastly, a 200 ns MD simulation was performed in an NPT ensemble at 300 K and 1 atm. Energy and atomic coordinate trajectory data were recorded every 48 ps.

### Disclaimer

Certain commercial materials and equipment are identified in order to adequately specify experimental procedures. Such identifications neither imply recommendation or endorsement by the National Institute of Standards and Technology nor do these imply that the material or equipment identified is the best available for the purpose.

## Electronic supplementary material

Supplementary Info
