# Supplementary material for: Effects of Distal Mutations on the Structure, Dynamics and Catalysis of Human Monoacylglycerol Lipase
Source: Sci Rep. 2018 Jan 29;8:1719. doi: 10.1038/s41598-017-19135-7 (PMC5789057; doi:10.1038/s41598-017-19135-7)

**Supplementary material for**

**Effects of Distal Mutations on the Structure, Dynamics and Catalysis of Human Monoacylglycerol Lipase**

Sergiy Tyukhtenko^1,6†^, Girija Rajarshi^1†^, Ioannis Karageorgos^2,3^, Nikolai Zvonok^1^, Elyssia S. Gallagher^2,3,4^, Hongwei Huang^‡5^, Kiran Vemuri^1^, Jeffrey W. Hudgens^2,3^, Xiayou Ma^1^, Mahmoud L. Nasr^6^, Spiro Pavlopoulos^1^ and Alexandros Makriyannis^1,6^

**^1^**Center for Drug Discovery and Departments of Pharmaceutical Sciences and Chemistry and Chemical Biology, Northeastern University, Boston, Massachusetts 02115-5000,

**^2^**BioProcess Measurements Group, Biomolecular Measurement Division, National Institute of Standards & Technology, Rockville, MD 20850,

**^3^**Institute for Bioscience and Biotechnology Research, 9600 Gudelsky Drive, Rockville, MD 20850,

^4^Department of Chemistry and Biochemistry, Baylor University, Waco, TX 76798,

### ^5^Schrödinger, 222 Third Street Suite 2230, Cambridge, MA 02142, and

### ^6^Department of Biological Chemistry and Molecular Pharmacology, Harvard Medical School, Boston, Massachusetts, 02115.

^†^The first two authors should be regarded as Joint First Authors

^‡^Present address: Dassault Systèmes, 9 Industrial Road, Milford, MA 01757

^6^To whom correspondence should be addressed: [a.makriyannis@northeastern.edu](mailto:a.makriyannis@northeastern.edu), [s.tyukhtenko@northeastern.edu](mailto:s.tyukhtenko@northeastern.edu)

**Figure S1.** Comassie blue stained SDS-PAGE analyses of purified enzymes expressed from E-coli BL 21(DE3) Cells. Lane 1, Molecular mass standards (kDa, Biorad); 2, sol-hMGL; 3, W289L; 4, W289F and 5, L232G.


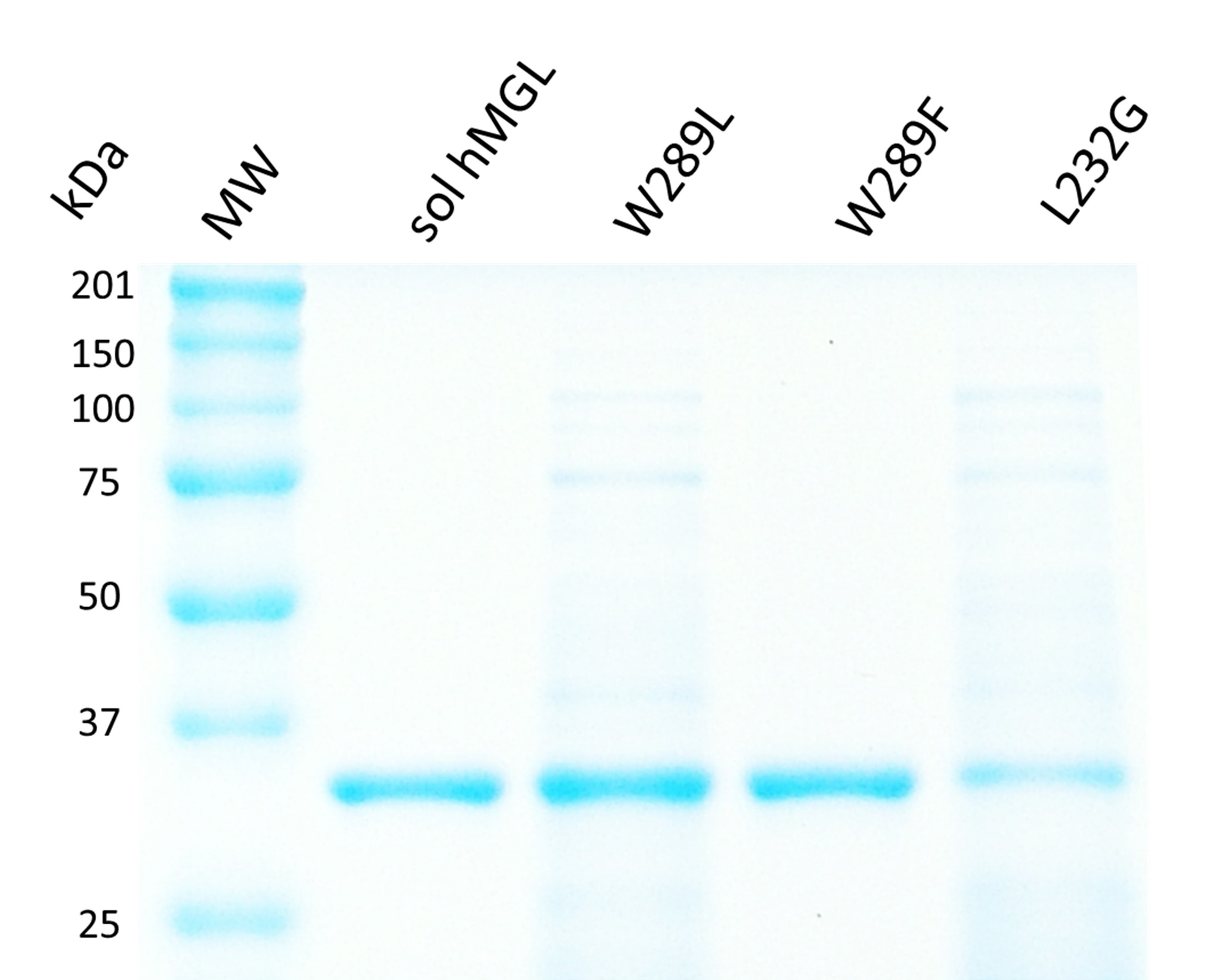


**Figure S2.** Substrate saturation curves obtained by fitting the reaction velocities in Michaelis- Menten equation for (a) sol-hMGL and tryptophan mutants W35A, W289L. (b) Zoomed-in graph demonstrating residual activities of L232G and W289L. Visible bars on each datum indicate 1σ measurement uncertainty; otherwise, 1σ uncertainties reside in the symbol.


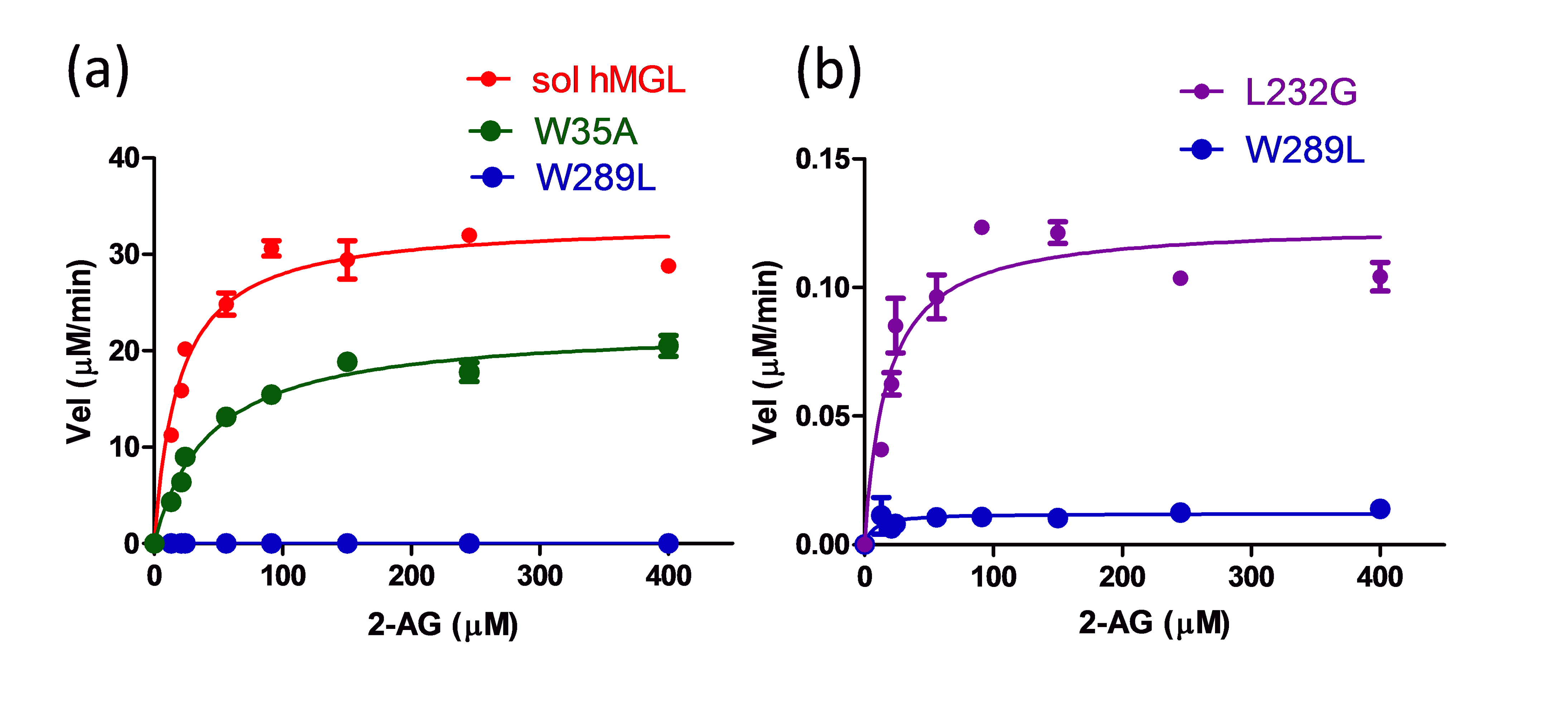


**Figure S3.** Urea-induced unfolding measurements in hMGL mutants, recorded by CD spectroscopy. The far-UV CD spectra of recombinant purified proteins (a) W289F and (b) W289L are compared with those being subjected to denaturation with 8 M urea.


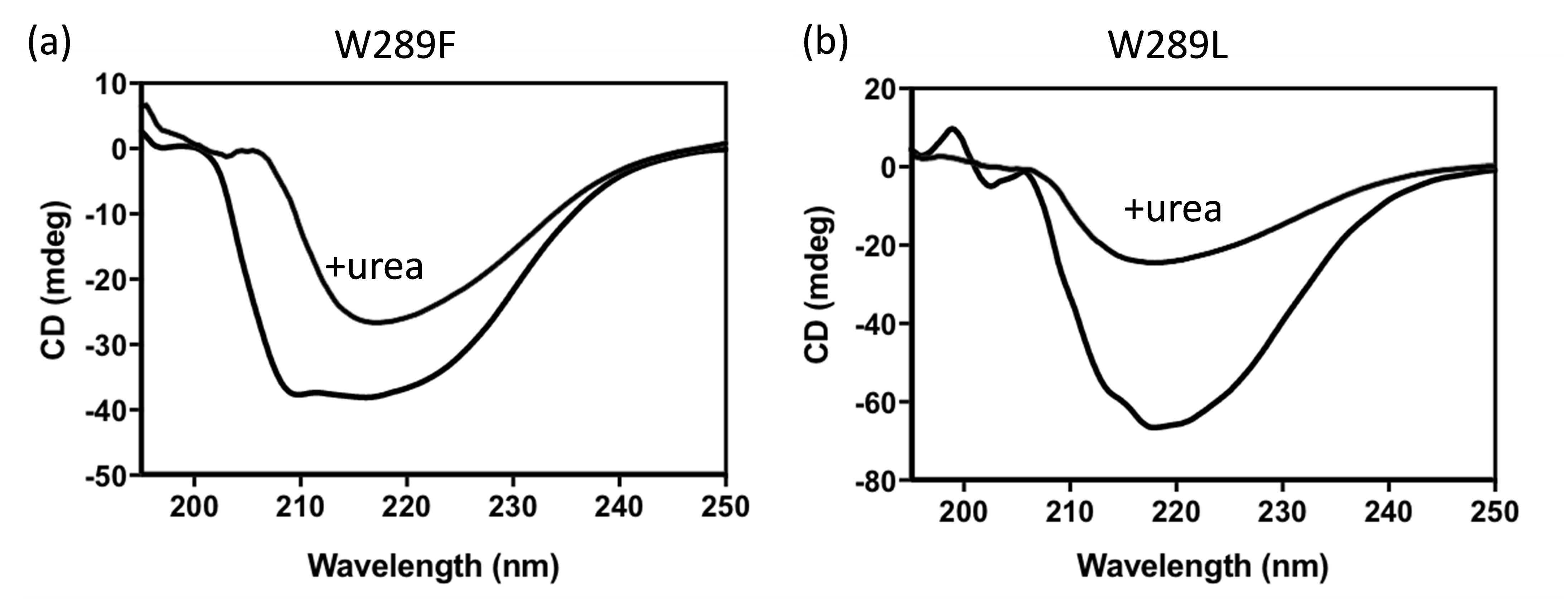


**Figure S4.** Folding of sol-hMGL (closed state), W289L, L232G and W289F (closed state) assessed by 1D ^1^H NMR data. (a) The downfield NMR resonances (17.5 – 9.7 ppm) originated from a tight tertiary packing of constructs. (b) The amide region (10.6 – 6.2 ppm) of the constructs. (c) upfield NMR resonances (0.7 - – 0.85 ppm) exhibiting patterns consistent with native tertiary packing.


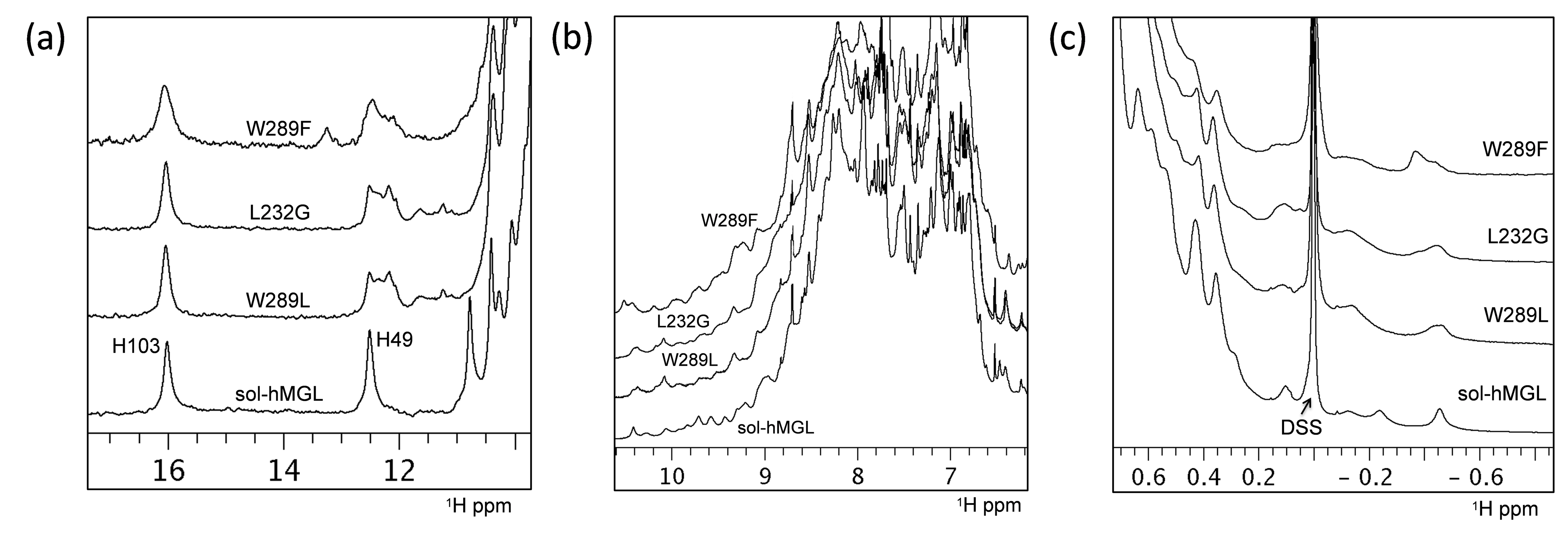


**Figure S5.** Sequence coverage map for peptic peptides that were identified by MS/MS spectra for: (a) wt hMGL and (b) W289L mutant. The peptides are presented as bars.


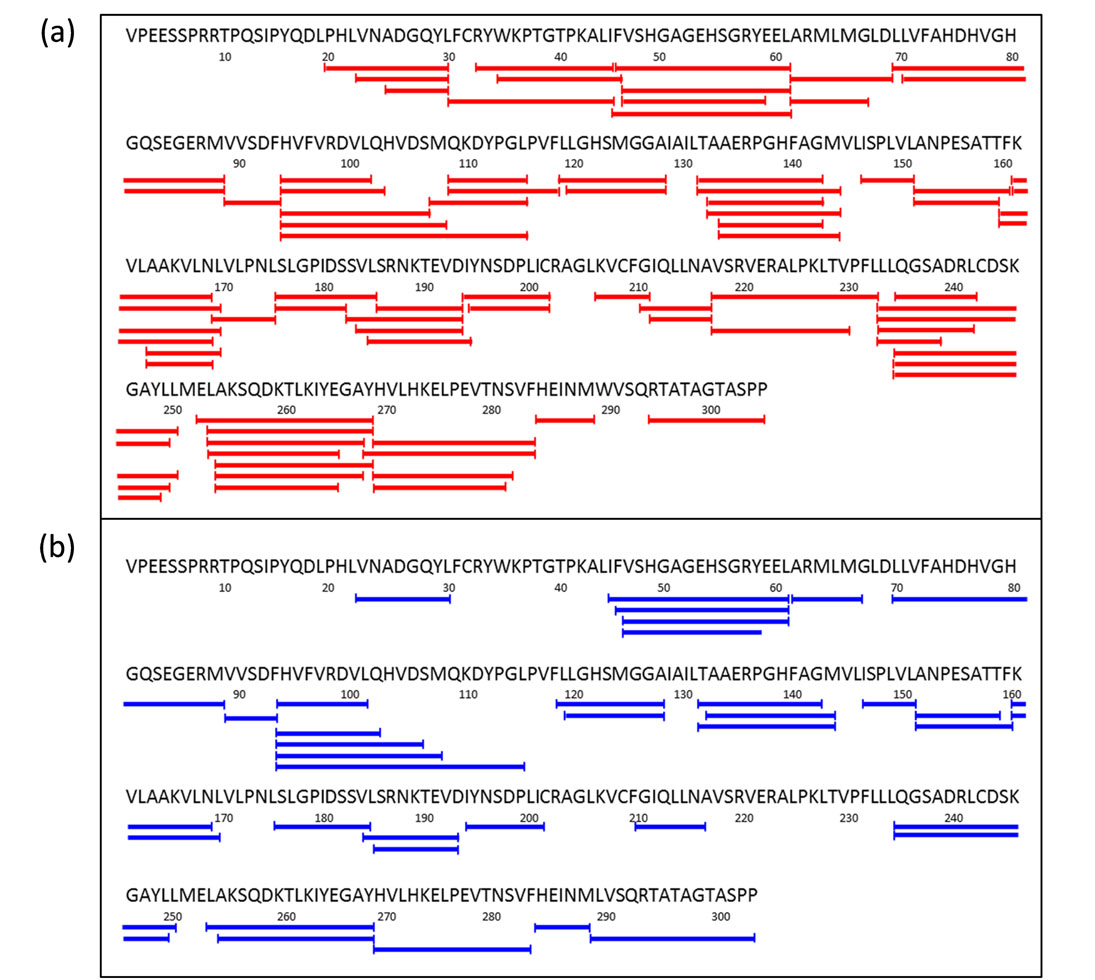


**Figure S6.** (a) RMSD evolution for C_α_ atoms of wt hMGL (red) and W289L (blue) during a 200 ns MD simulation. (a) The calculated R.M.S.F. values for the C_α_ atoms of wt hMGL and W289L with respect to the initial conformation (open) during the 200 ns MD simulations. The residues comprising the lid domain are highlighted.


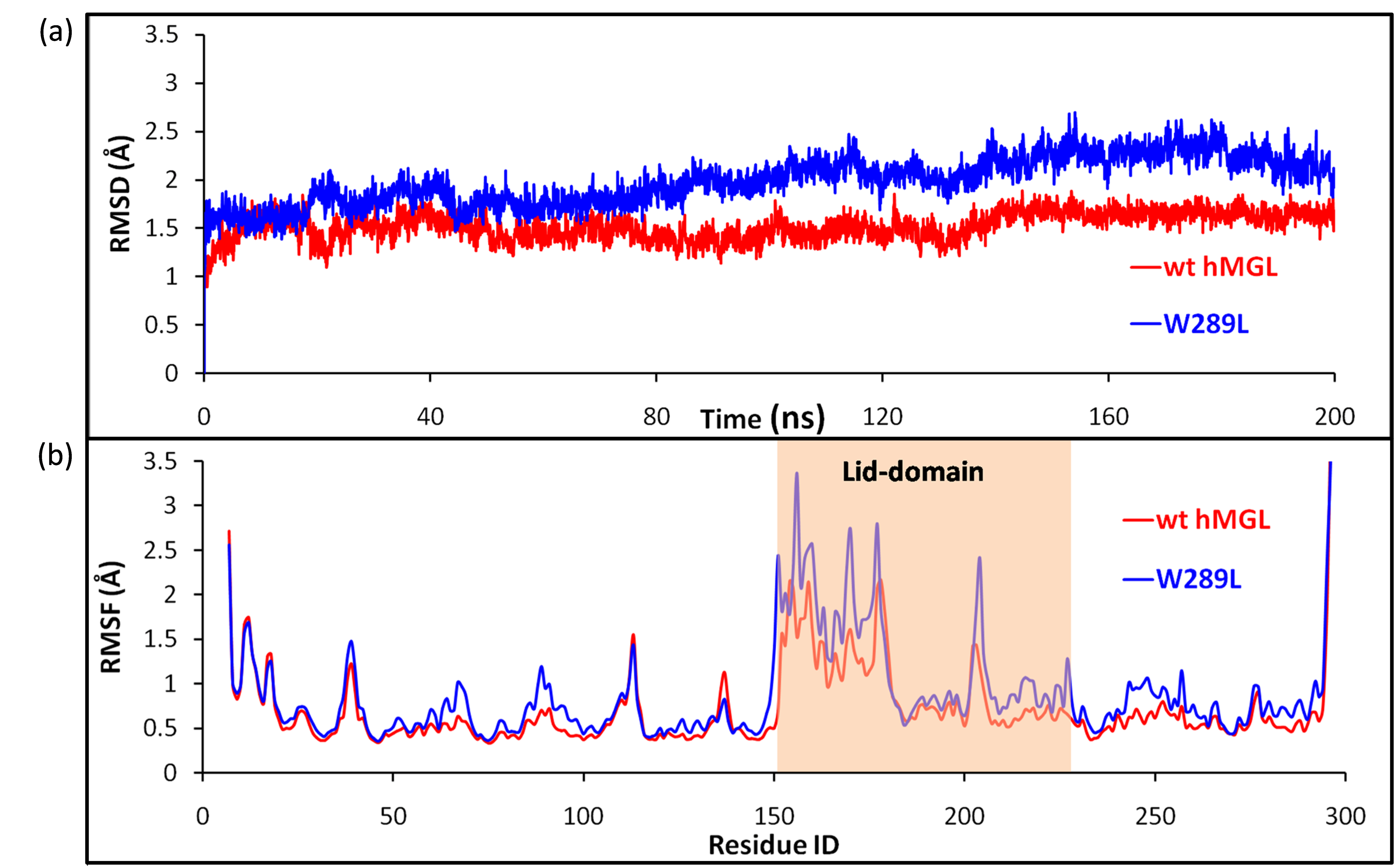


**Figure S7.** RMSD evolution for Cα atoms of wt hMGL (red) and W289L (blue) for (a) Residues comprising the lid-domain (151-225) and (b) Residues comprising the core of the protein, during 200ns MD simulation time.


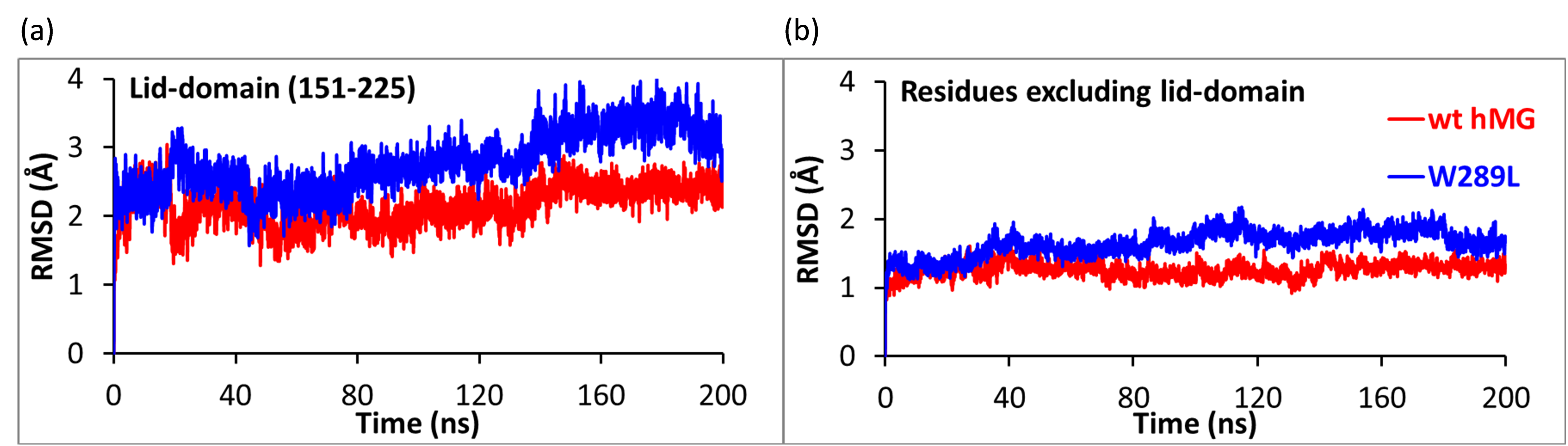


**Figure S8.** RMSD evolution for Cα atoms of wt hMGL (red) and W289L (blue) during 200ns MD simulation time compared to closed conformation (PDB ID: 3PE6)


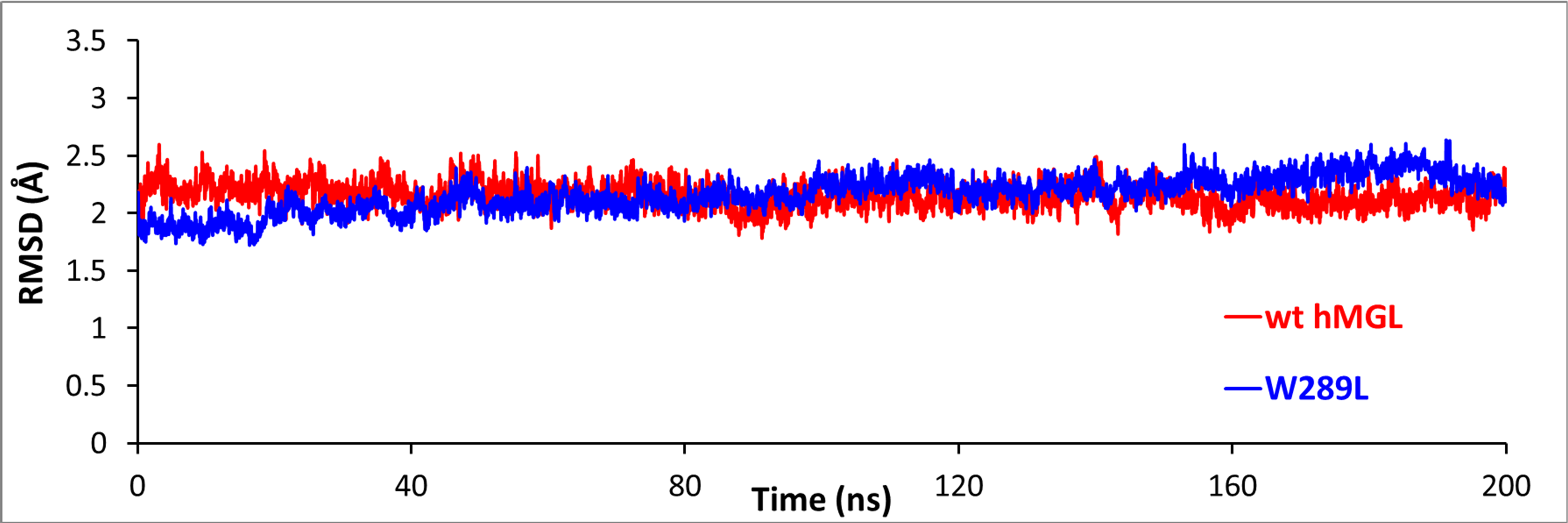


**Figure S9.** The calculated R.M.S.F. values for the Cα atoms of wt hMGL (red) and W289L (blue) with respect to the average structure during the last 100ns MD simulations. The residues comprising the lid domain are highlighted.


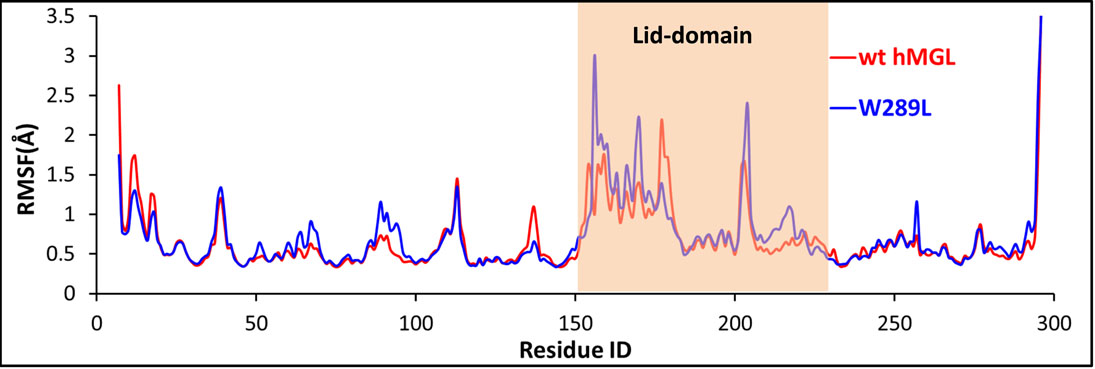


**Figure S10.** The distance between C_α_ atoms of Phe159 and Gly210 for wt hMGL (red) and W289L (blue) for MD simulations of 200 ns duration.


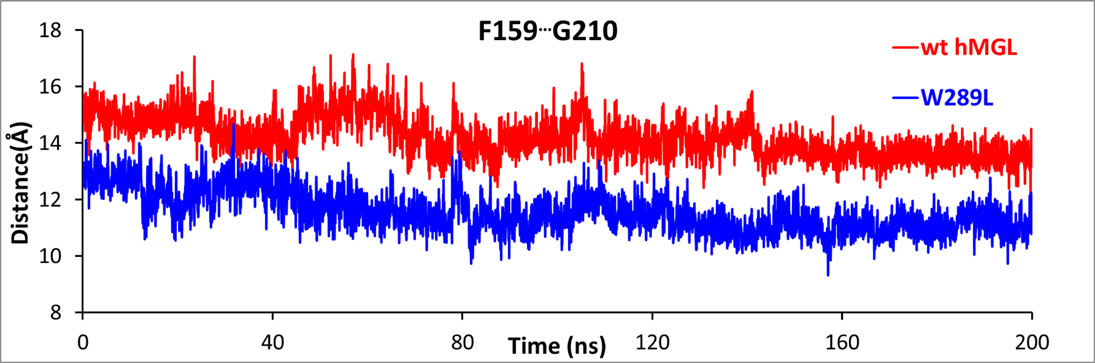

Supplement: Supplementary file 1 — Supplementary Info [file 41598_2017_19135_MOESM1_ESM.doc]
